# 3D Porous Oxygen‐Doped and Nitrogen‐Doped Graphitic Carbons Derived from Metal Azolate Frameworks as Cathode and Anode Materials for High‐Performance Dual‐Carbon Sodium‐Ion Hybrid Capacitors

**DOI:** 10.1002/advs.202301160

**Published:** 2023-06-16

**Authors:** Yong Min Jung, Jong Hui Choi, Dong Won Kim, Jeung Ku Kang

**Affiliations:** ^1^ Department of Materials Science and Engineering Nano Century Institute Korea Advanced Institute of Science and Technology (KAIST) 291 Daehak‐ro, Yuseong‐gu Daejeon 34141 Republic of Korea

**Keywords:** dual‐carbon sodium‐ion hybrid capacitors, high energy densities, fast chargeable power densities, and robust cycle stabilities, metal‐azolate frameworks, porous nitrogen‐doped graphitic carbon anodes, ultrahigh surface area porous oxygen‐doped graphitic carbon cathodes

## Abstract

Sodium‐ion hybrid capacitors (SIHCs) in principle can utilize the advantages of batteries and supercapacitors and satisfy the cost demand of large‐scale energy storage systems, but the sluggish kinetics and low capacities of its anode and cathode are yet to be overcome. Here, a strategy is reported to realize high‐performance dual‐carbon SIHCs using 3D porous graphitic carbon cathode and anode materials derived from metal–azolate framework‐6s (MAF‐6s). First, MAF‐6s, with or without urea loading, are pyrolyzed to synthesize MAF‐derived carbons (MDCs). Then, cathode materials are synthesized via the controlled KOH‐assisted pyrolysis of MDCs (K‐MDCs). K‐MDCs, 3D graphitic carbons, resulting in a record‐high surface area (5214 m^2^ g^−1^) being ≈four‐fold higher than pristine MAF‐6, oxygen‐doped sites for high capacity, rich mesopores affording fast ion transport, and high capacity retention over 5000 charge/discharge cycles. Moreover, 3D porous MDC anode materials are synthesized from N‐containing MAF‐6 and exhibited to allow cycle stability over 5000 cycles. Furthermore, dual‐carbon MDC//K‐MDC SIHCs with different loadings (3 to 6 mg cm^−2^) are demonstrated to achieve high energy densities exceeding those of sodium‐ion batteries and supercapacitors. Additionally, it allows an ultrafast‐chargeable high power density of 20000 W kg^−1^ and robust cycle stability overcoming those of a typical battery.

## Graphical abstract

It is developed the 3D porous oxygen‐doped and nitrogen‐doped graphitic carbons derived from metal azolate frameworks as the cathode and anode materials for high‐performance dual‐carbon sodium‐ion hybrid capacitors, as demonstrated by a high energy density exceeding those of sodium‐ion batteries and supercapacitors, an ultrafast‐chargeable power density (20000 W kg^−1^) overcoming the limitation of a battery, and robust cycle stability.

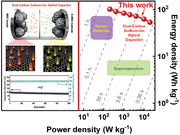

## 1 Introduction

The requirement for high‐performance electrochemical energy storage systems (EESs) has increased gradually with the growing economy and increasing living standards.^[^ ^1^, ^2^, ^3^ ^]^ Moreover, hybrid capacitors have been developed to utilize the advantages of batteries and supercapacitors.^[^ ^4^, ^5^ ^]^ Especially, sodium‐ion hybrid capacitors (SIHCs) have attracted considerable attention to replace conventional lithium‐ion hybrid capacitors (LIHCs) considering the low cost and abundant availability of sodium compared with lithium.^[^ ^6^, ^7^ ^]^ Furthermore, the search for SIHCs affording high energy density and fast chargeable power density, as well as robust cycle stability is of worldwide efforts. However, there remains a critical challenge to balance the charge and reaction kinetics between their cathodes and anodes with low capacity, sluggish ion diffusion, and poor electron conductivity. This mismatch between anode and cathode materials is still a big hurdle to achieving high‐performance SIHCs.^[^ ^8^ ^]^ Utilization of similar materials, such as carbons for the cathode and anode electrodes of SIHCs could be a possible way to overcome this challenge.^[^ ^9^ ^]^

It is important to find hierarchical porous cathode and anode materials allowing reversible and rapid cation adsorption/desorption and anion intercalation/deintercalation capability with abundant active sites for large capacity. However, the sluggish redox reaction kinetics in an anode material, originating from the bigger ionic radius of Na^+^ (1.02 Å) compared with that of Li^+^ (0.76 Å), is a problem. Various materials, such as metal oxides/sulfides/selenides and carbons have been investigated as the anode materials of sodium‐ion energy storages.^[^ ^10^, ^11^, ^12^, ^13^, ^14^, ^15^, ^16^, ^17^, ^18^, ^19^, ^20^, ^21^, ^22^ ^]^ Among them, carbonaceous materials are promising candidates for the anodes of SIHCs because they have superior electron/ion conductivity and robust cycle stability.^[^ ^23^ ^]^ Besides, several strategies, such as heteroatom‐doping and porous structure‐tuning have been developed.^[^ ^24^ ^]^ These methods were proven to result in high capacity and good cycle stability. However, the energy density of a SIHC was found to be still limited owing to the low specific capacity of its counter cathode material.^[^ ^25^ ^]^ A porous carbon is generally widely used as a cathode material of a SIHC. For example, activated carbon or graphene was studied as the cathode of a SIHC, but it has a low specific capacity (30–50 mAh g^−1^) attributed to the weak chemical binding between graphene and Na.^[^ ^26^ ^]^ Moreover, the exploration of a hierarchically porous cathode material with abundant diffusion channels is very important to improve the electrochemical performances of a SIHC. Especially, abundant interconnected open mesopores are crucial to enable both fast ion transport and also full‐capacity harvesting even from a high surface area cathode material affording rich redox‐active sites for anions. Hence, developing anode and cathode materials that can overcome these challenges is essential to realize high‐performance SIHCs.

A metal–organic framework (MOF) is synthesized through the formation of a coordination bond between metal ions/clusters and organic ligands,^[^ ^27^ ^]^ so that its high porosity and tunable physiochemical properties lead to advanced functionality for various applications, such as gas storage/separation,^[^ ^28^, ^29^ ^]^ energy storage,^[^ ^30^, ^31^ ^]^ and catalysis.^[^ ^32^ ^]^ Moreover, MOFs have attracted tremendous attention because of their tailored structure allowing tunable chemical properties.^[^ ^33^, ^34^, ^35^, ^36^, ^37^ ^]^ In this work, we utilized a metal azolate framework‐6 (MAF‐6) with the pore aperture of 7.6 Å and the pore diameter of 18.0 Å, which is composed of Zn^2+^ ions and 2‐ethylimidazole ligands, to synthesize a high‐capacity and high‐rate cathode structure for a SIHC. MAF‐6 is an interesting MOF because its large pore aperture and pore size are effective for loading extra species. The carbonization of MAF‐6s after loading urea in a suitable quantity led to functional carbons. Also, the synthesis by controlling the N‐content of MAF‐6‐derived carbons, in the presence of KOH is shown to result in 3D ultrahigh porous oxygen‐doped graphitic carbons, implying that chemical modification with additional activators after the primary carbonization of MAF‐6 is effective to control both the porosity and pore size of the obtained carbon. Moreover, MAF‐6s were successfully converted into 3D porous nitrogen‐doped graphitic carbons as anode materials, where N‐containing ligands provide nitrogen doping effect on anode materials thereby allowing both excellent electrolyte wettability and also abundant pseudocapacitive redox‐reaction sites for high capacity. The preparation of the 3D porous oxygen‐doped graphitic carbon cathode and nitrogen‐doped graphitic carbon anode electrodes from the same precursor (MAF‐6) is simple, convenient, and attractive. Also, using the carbons derived from the same precursor in the two electrode materials for a SIHC is very effective to reduce the mismatch between cathode and anode materials. Furthermore, we demonstrate that the 3D porous oxygen‐doped graphitic and nitrogen‐doped graphitic carbons derived from MAF‐6s can be utilized as the cathode and anode materials to realize remarkable performances in energy density, power density, and cycle stability in a dual‐carbon SIHC full‐cell configuration.

## 2 Results and Discussion

**Figure 1:**
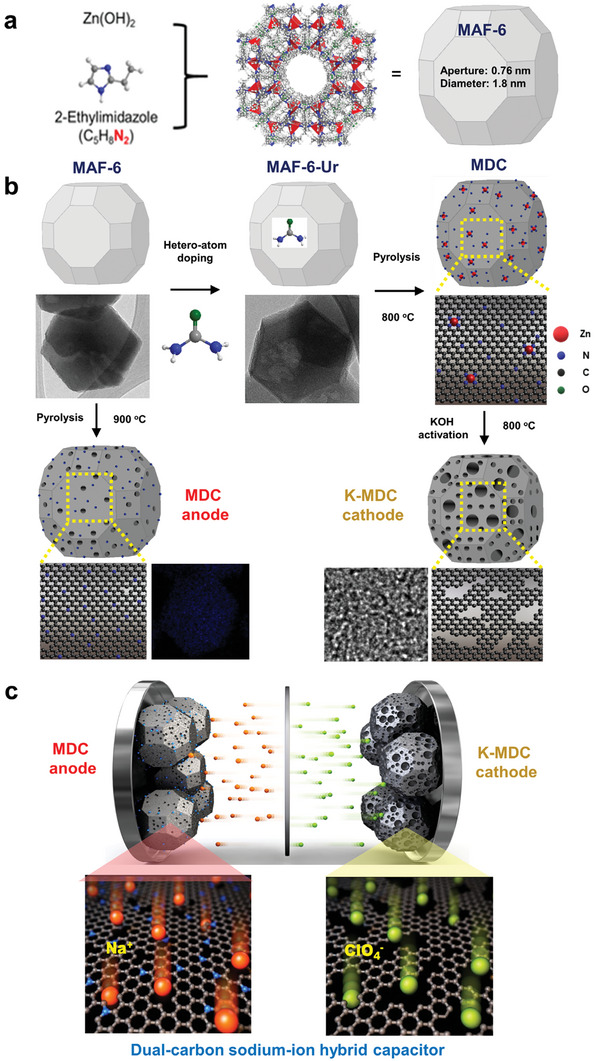
Schematic illustration of the synthetic processes and configurations of a dual‐carbon sodium‐ion hybrid capacitor (SIHC). a) Schematics for the synthesis of a large‐pore metal–azolate framework, MAF‐6 (pore aperture: 0.76 nm, pore diameter: 1.8 nm) from Zn precursor and 2‐ethylimidazole linker. b) Schematics for the synthesis of nitrogen‐doped porous carbon (MDC) anode and ultrahighly porous carbon (K‐MDC) cathode materials from MAF‐6 and urea‐loaded MAF‐6. c) Schematic energy storage mechanism in MDC anode and K‐MDC cathode of a dual‐carbon SIHC.

The whole synthetic processes and SIHC configuration are illustrated in **Figure** **1**. First, MAF‐6 was prepared from Zn precursor and 2‐ethylimdazole ligand (Figure 1a). The synthesis processes of cathode and anode materials from MAF‐6 are presented in Figure 1b. An optimized amount of urea was loaded in the pores of MAF‐6 to prepare MAF‐6‐Ur20. Then, Zn—N containing nonporous carbon, MDC(20), was obtained by pyrolysis of MAF‐6‐Ur20 at 800 °C. After that, K‐MDC(20), which is a highly porous carbonaceous material, was prepared by KOH activation of MDC(20). The finally obtained K‐MDC was adopted as a cathode material. Simultaneously, MAF‐6 was converted to MDC structure (anode) by a simple pyrolysis without further modification. The obtained MDC anode with rhombohedral dodecahedrons (similar to the MAF‐6 precursor), which is a nitrogen‐doped porous carbon, was utilized as the anode material of a SIHC. Furthermore, the schematic illustration of MDC//K‐MDC full cell and the corresponding energy storage mechanism between the electrolyte and electrode are presented in Figure 1c. The synthesized MAF‐6 was characterized by X‐ray diffraction (XRD) and N_2_ adsorption analyses. As illustrated in Figure S1a (Supporting Information), the XRD pattern of MAF‐6 was in good agreement with that of the calculated one based on the crystal structure. Furthermore, the N_2_ adsorption isotherm of MAF‐6 in Figure S1b (Supporting Information) was determined to be similar to those of the reported results.^[^ ^38^ ^]^ Based on the above results, the successful synthesis of MAF‐6 could be confirmed. The whole procedure to optimize cathode material, K‐MDCs, from the synthesized MAF‐6 is schematically illustrated in Figure S2 (Supporting Information). First, pristine MAF‐6 and MAF‐6s with urea loading in different amounts (MAF‐6‐Ur20 and MAF‐6‐Ur50, 20, and 50) indicate the percentages of loaded urea against the weight of MAF‐6), were prepared. The three types of MAF‐6 samples were pyrolyzed to obtain MDC(0), MDC(20), and MDC(50). The content of Zn atoms in carbonaceous materials increased with the increasing amount of loaded urea, which was found to be attributed to the strong interaction between Zn and N. Through the chemical activation of these MDCs, the three porous carbons with different porous structures (K‐MDC(0), K‐MDC(20), and K‐MDC(50)) were prepared via the removal of heteroatoms (especially Zn—N) that existed in MDCs. These K‐MDCs were electrochemically tested as the cathode materials of SIHCs. MAF‐6s pyrolyzed in the first step were characterized by XRD, X‐ray photoelectron spectroscopy (XPS), N_2_ adsorption, etc. As presented in Figure S3 (Supporting Information), all three carbons, MDC(0), MDC(20), and MDC(50), had the broad XRD patterns at 2*θ* of 23° and 43°, suggesting that the carbons are composed of disordered carbons (quartz‐free) with a graphitic phase. The XPS spectra were further utilized to explore the surface composition of MDCs. Figure S4a (Supporting Information) shows that all three carbons are composed of similar atoms, such as C, O, and N. Interestingly, the Zn and Cl contents of MDCs increased with the increasing loaded urea content in MAF‐6 before pyrolysis. Therefore, the very little porosity of MDC(20) and MDC(50) can be explained by the remaining Zn and Cl species in the two MDCs. Moreover, the XPS spectra for N species in MDCs were deconvoluted to determine the chemical status of the remaining zinc atoms (Figure S4b, Supporting Information). N 1*s* spectra can be deconvoluted with Zn—N, pyridinic, pyrrolic, graphitic, and oxidized N as presented in Table S1 (Supporting Information); the contributions of Zn—N to the total N of MDC(0), MDC(20), and MDC(50) are 1%, 14%, and 25%, respectively. Figure S5a (Supporting Information) also shows the N_2_ adsorption isotherm of MDC(0). After the pyrolysis of MAF‐6 at 800 °C, a porous carbon was obtained. The isotherm is type I, supporting that MDC(0) is a microporous material, which is in agreement with the pore size distribution (PSD) in Figure S5b (Supporting Information). However, interestingly, MDC(20) and MDC(50) adsorb very little N_2_, different from MDC(0). Brunauer–Emmett–Teller (BET) surface areas are 1003, 48, and 31 m^2^ g^−1^ for MDC(0), MDC(20), and MDC(50), respectively. Additionally, the high‐angle annular dark‐field (HAADF)‐scanning transmission electron microscopy (STEM) and energy dispersive spectroscopy (EDS)‐mapping images of MDC(20) in Figure S6 (Supporting Information) show that heteroatoms (N, Zn, Cl, and O) are homogeneously distributed in a carbon structure.

**Figure 2:**
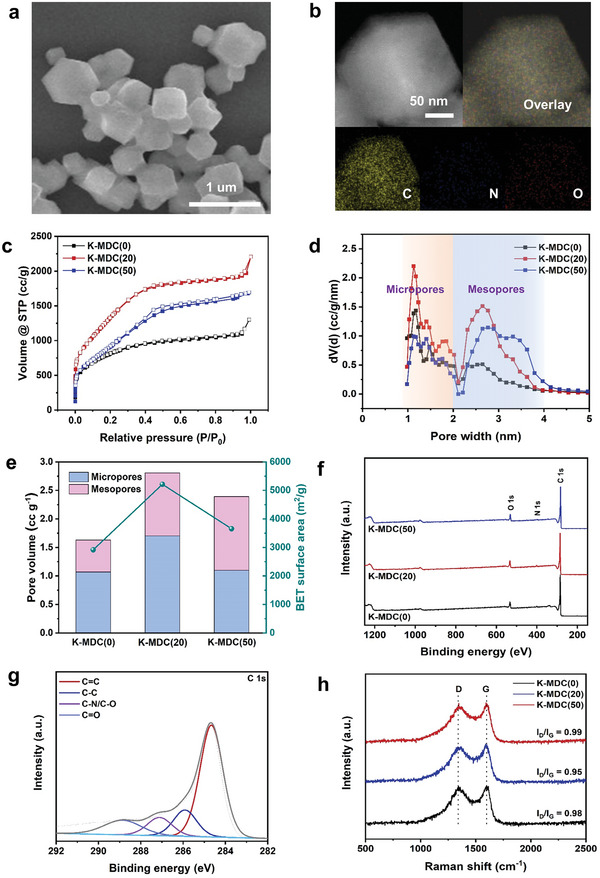
Structural characterizations of K‐MDCs. a) SEM image of K‐MDC(20). b) HAADF‐STEM and EDS‐mapping images of K‐MDC(20). c) N_2_ adsorption–desorption isotherms and d) corresponding pore size distributions of K‐MDCs. e) Comparison of pore volumes and BET surface areas of K‐MDCs. f) XPS spectra of K‐MDCs and g) high‐resolution C 1*s* spectrum of K‐MDC(20). h) Raman spectra of K‐MDCs.

K‐MDCs were also characterized through XRD, scanning electron microscope (SEM)/TEM, N_2_ adsorption, XPS, and Raman spectroscopy analyses. After the KOH activation of MDCs, the (002) peaks of MDCs at 2*θ* of 23° became very weak and broad, suggesting the formation of highly amorphized carbons (Figure S7, Supporting Information). SEM/TEM images in **Figure** **2**a and Figure S8 (Supporting Information) confirm that there is little change in morphology upon the urea loading and pyrolysis of MAF‐6 to K‐MDC(20). Furthermore, the EDS mappings of K‐MDC(20) in Figure 2b confirm that C, N, and O atoms are evenly distributed throughout the nanocrystal. As presented in Figure 2c, the N_2_ adsorption of K‐MDC(0) reveals that the material is mainly composed of micropores. K‐MDC(20) and K‐MDC(50) showed the BET surface areas of 5214 and 3657 m^2^ g^−1^, respectively. Additionally, the PSDs (Figure 2d), derived from the isotherms, show that K‐MDC(20) has mesopores with a pore size of 2–4 nm. As compared in Figure 2e, the volume ratio of mesopore/total pore increases in the following order: K‐MDC(0) < K‐MDC(20) < K‐MDC(50). The whole textural properties of MDCs and K‐MDCs are summarized in Table S2 (Supporting Information). The BET surface area of K‐MDC(20) is one of the highest results observed among carbonaceous materials, especially from the pyrolysis of MOFs. It is very interesting how non‐porous MDC(20) can be converted into very highly porous carbon by the second‐step KOH activation under 800 °C, as illustrated in Figure S9 (Supporting Information). During KOH activation, carbons are removed via the formation of CO and CO_2_, leading to the formation of pores. The strong interaction between Zn and N via Zn—N bonding, as revealed in the XPS results in Figure S4b (Supporting Information), has a crucial role in the generation of porosity, especially in the mesopore region. However, too many Zn/N species cause the destruction of pores leading to larger pores and low porosity, as confirmed by the BET surface area and pore volumes of K‐MDC(50) are lower than those of K‐MDC(20). XPS spectra in Figure 2f and Table S3 (Supporting Information) present that the carbons have similar surface compositions (mainly C with slight O and N) without Zn species. The carbon atoms of K‐MDC(20) are mainly composed of C=C/C—C with slight C—O/C—N/C=O, as shown in Figure 2g. Finally, all carbons of K‐MDCs are graphitic and defective, as presented in the Raman spectra of Figure 2h.

**Figure 3:**
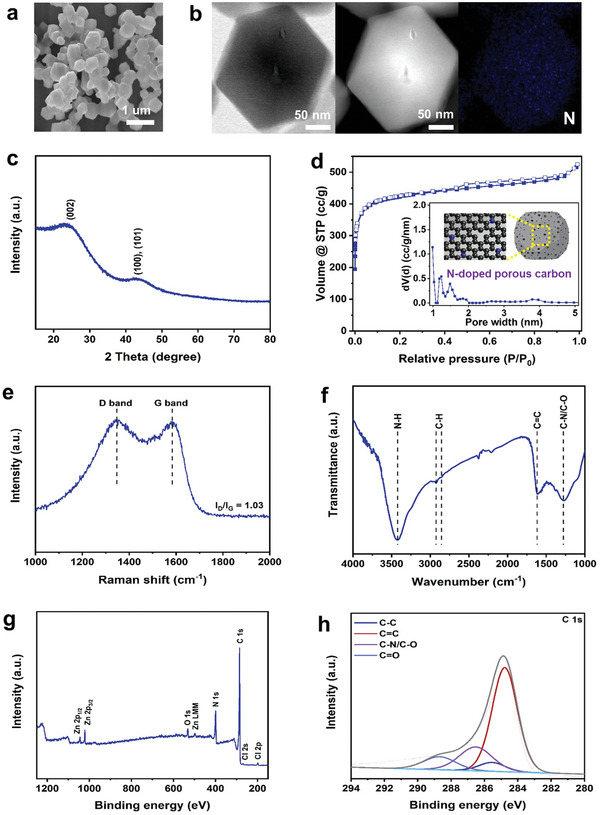
Structural characterizations of MDC anode. a) SEM image, b) TEM, HAADF‐STEM, and EDS‐mapping images, c) XRD patterns, d) N_2_ adsorption–desorption isotherm (inset: pore size distribution and schematic illustration of MDC anode), e) Raman spectra, f) FTIR spectra, g) XPS spectra, and h) high‐resolution C 1*s* XPS spectra.

The SEM image (**Figure** **3**a) supports that MDC anode material, prepared via one‐step pyrolysis of MAF‐6 at 900 °C, is composed of rhombohedral dodecahedrons. The characteristic structure and composition of MDC anode material were further analyzed through TEM, HADDF‐STEM, and nitrogen EDS‐mapping images (Figure 3b). The agglomeration of particles was not observed after carbonized procedure, from the HADDF‐STEM results. Moreover, the homogeneously distributed nitrogen heteroatoms throughout the porous structure of MDC anode material were confirmed by TEM‐EDS mapping. The two broad peaks at 2*θ* of 23° and 43° were observed in the XRD pattern of MDC anode material, as exhibited in Figure 3c. This demonstrates that the carbon has a disordered graphite character. The N_2_ sorption isotherm with the corresponding PSD result (Figure 3d) and Table S4 (Supporting Information) suggest the porous structure of the anode material. The nitrogen‐doped porous carbon structure is schematically illustrated to emphasize the characteristic structure of MDC. Besides, Raman spectrum (Figure 3e) supports that MDC anode material is composed of defective and graphitic carbons with the corresponding intensity ratio of 1.03 for D band to G band. The FTIR spectra of this nitrogen‐doped carbon (Figure 3f) suggest further the presence of N—H and C—N/C—O bonds. Moreover, XPS analysis in Figure 3g confirms the existence of N element in MDC anode material. O, Zn, and Cl, as well as C and N were observed from the XPS survey results, as shown in Table S5 (Supporting Information). Zn species were originated from the pristine MAF‐6 composed of Zn^2+^ ions. It is noteworthy that the proper carbonized temperature (900 °C) to synthesize MDC anode material is lower than the boiling point of zinc, 907 °C. During the soaking procedure to remove residual zinc species by using a concentrated HCl solution, Cl ions could be doped as heteroatoms to carbonaceous materials, as verified by XPS spectra (Figure 3g). Meanwhile, the contents of Cl ions are negligible. Moreover, the deconvoluted C 1*s* XPS spectra (Figure 3h) reveal the existence of C—N/C—O bond in MDC anode material, similar with FTIR spectra.

**Figure 4:**
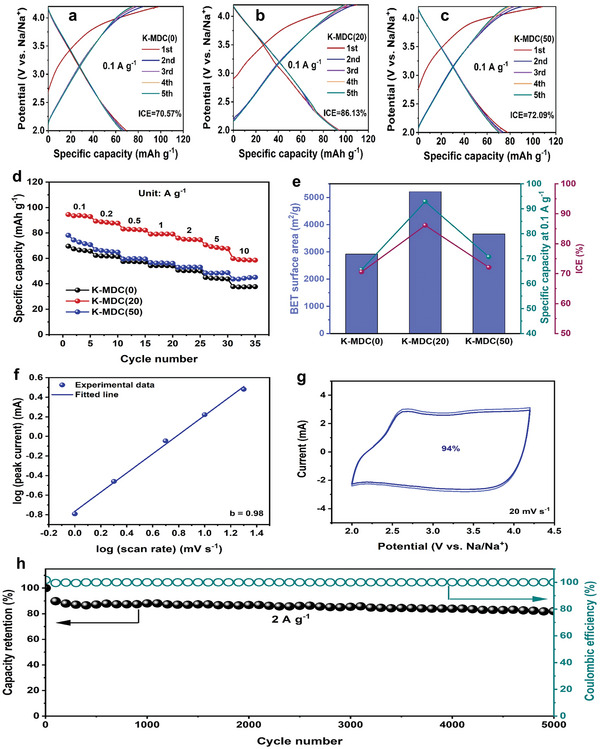
Electrochemical performances of K‐MDC cathode. GCD profiles of a) K‐MDC(0), b) K‐MDC(20), and c) K‐MDC(50) at 0.1 A g^−1^. d) Rate performances of K‐MDCs. e) Relation between the electrochemical performances and BET surface area of K‐MDCs. f) Plot of log (current) versus log (scan rate) from peak currents. g) Capacitive contribution of K‐MDC(20) at a scan rate of 20 mV s^−1^. h) Cycling stability of K‐MDC(20) at 2 A g^−1^.

The electrochemical properties of cathode materials were evaluated within the potential range of 2.0–4.2 V (vs. Na/Na^+^). The cyclic voltammetry (CV) curves of K‐MDCs in Figures S10 and S11 (Supporting Information) at the scan rate of 1–20 mV s^−1^ present a quasi‐rectangular shape with slight humps, which is well maintained at any scan rate. The observed shape of the curves suggests a pseudocapacitive behavior, which is attributed to the oxygen species.^[^ ^39^ ^]^ Moreover, the galvanostatic charge/discharge (GCD) curves at 0.1 A g^−1^ during five cycles are shown in **Figure** **4**a–c. Polarizations were observed in the first GCD curves of K‐MDCs, which are originated from the side effects related to oxygen heteroatoms, residual impurities and waters, and electrochemical activation procedures.^[^ ^40^ ^]^ Meanwhile, the GCD curves of the following cycles were almost overlapped, which indicates the reversible and stable reactions of K‐MDCs. The reversible capacity of K‐MDC(20) at 0.1 A g^−1^ is 93 mAh g^−1^, superior to those of K‐MDC(0) (65 mAh g^−1^) and K‐MDC(50) (71 mAh g^−1^). This suggests that the significant improvement in the electrochemical performance of K‐MDC(20), compared with that of pristine MAF‐6 derived K‐MDC(0), is attributed to the urea loading to MAF‐6. However, as presented in the results of K‐MDC(50), the specific capacity decreases when the excess urea is loaded into MAF‐6. Besides, the initial coulombic efficiencies (ICEs) of K‐MDC(0), K‐MDC(20), and K‐MDC(50), are 70.6%, 86.1%, and 72.1%, respectively. This suggests that the different ICEs are attributed to the different porous structures or porosities of the carbons. In other words, the ultrahigh surface area and huge pore volume of K‐MDC(20) lead to the reduced ion diffusion pathway, resulting in the acceleration of ion diffusion and reversible electrochemical reaction. Additionally, the GCD curves of all K‐MDCs in Figures S12 and S13 (Supporting Information), which were obtained under various current densities (from 0.1 to 10 A g^−1^) were observed to be linear, suggesting electric double‐layer behavior consistent with the CV results. K‐MDC(20) presents the highest specific capacities among the three K‐MDCs. In addition, the changes in the specific capacities upon adjusting the current density from 0.1 to 10 A g^−1^ were evaluated to compare rate performances, as summarized in Figure 4d and Figure S14 (Supporting Information). Remarkably, we find that K‐MDC(20) leads to a reversible capacity of 59 mAh g^−1^ even under a high current density of 10 A g^−1^, suggesting an outstanding rate capability of K‐MDC(20). Figure 4e illustrates the relation between the specific surface area and electrochemical performances, such as ICE and specific capacity. Both the ICE and specific capacity values of K‐MDCs were found to be dependent on the BET surface area of the cathode materials, suggesting the importance of the porosity of the carbons derived through the chemical activation of MDCs. These results imply that K‐MDC(20) could play as a high‐capacity/high‐rate cathode material for SIHCs. To further elucidate the kinetics of K‐MDC(20), we analyzed the relationship between current (*i*) and scan rate (*υ*),^[^ ^41^ ^]^ as rewritten by

(1) $$i=a\upsilon^{b}\;\left(a\;\mathrm{and}\;b\;\mathrm{are}\;\mathrm{constants}\right)$$

As depicted in Figure 4f, the plot of log(*i*) versus log(*υ*) was obtained for K‐MDC(20) based on the peak currents from the CV curves (Figure S10, Supporting Information). The *b* value calculated from the slope of log(*i*) versus log(*υ*) can be used to classify the electrochemical processes. A *b* value close to 0.5 and 1 indicates that the charge storage mainly originates from a diffusion‐controlled process and surface‐induced capacitive process, respectively. The obtained *b* value from the fitting of experimental data of K‐MDC(20) is 0.98, which is very close to 1, demonstrating that the capacitive process is dominant. The high ratio of the capacitive process results in fast kinetics.^[^ ^42^ ^]^ Therefore, the outstanding rate capability of K‐MDC(20), is obtained. Moreover, the current *i* at a specific potential (V) can be divided into the following two terms^[^ ^43^ ^]^ of

(2) $$i\left(V\right)=k_{1}\upsilon +k_{2}\upsilon^{1/2}$$

where $k_{1}\upsilon$ and $k_{2}\upsilon^{1/2}$ represent the surface capacitive‐process and diffusion‐controlled process, respectively. The relative contributions of capacitive and diffusion‐controlled processes at various scan rates were summarized in Figure S15 (Supporting Information). The surface‐controlled capacitive contribution to the current was much higher than that of the diffusion‐controlled process irrespective of the scan rate. Moreover, the capacitive contribution gradually increased with the increasing scan rate. For example, the capacitive contribution was 94% at a high scan rate of 20 mV s^−1^, which is emphasized in Figure 4g. Therefore, the superior electrochemical property of K‐MDC(20) was mainly attributed to the capacitive process. This remarkably high capacitive contribution in K‐MDC(20) is attributed to the heteroatom doping and suitable pore size for electrolyte anions (ClO_4_^−^).^[^ ^44^ ^]^ These results demonstrate that the major charge storage of the electrochemically outstanding material, K‐MDC(20), results from a pseudocapacitive process allowing fast kinetics. Furthermore, the cycling stability of K‐MDC(20) was evaluated at 2 A g^−1^, as depicted in Figure 4h. The capacity of K‐MDC(20) did not decline significantly during 5000 cycles with a capacity retention of 82%, suggesting a superior cyclic performance of K‐MDC(20). The outstanding capacity retention originates from the facilitated kinetic process in the capacitive energy storage mechanism.^[^ ^45^ ^]^ Additionally, Table S6 (Supporting Information) shows the high reversible capacities and cyclic stability of K‐MDC(20) and cutting‐edge cathode structures.^[^ ^3^, ^18^, ^46^, ^47^ ^]^ Consequently, the high surface area, excellent reversible capacity, and robust cycle stability signal that K‐MDC(20) can be a very competitive cathode material to realize high performances in SIHCs.

**Figure 5:**
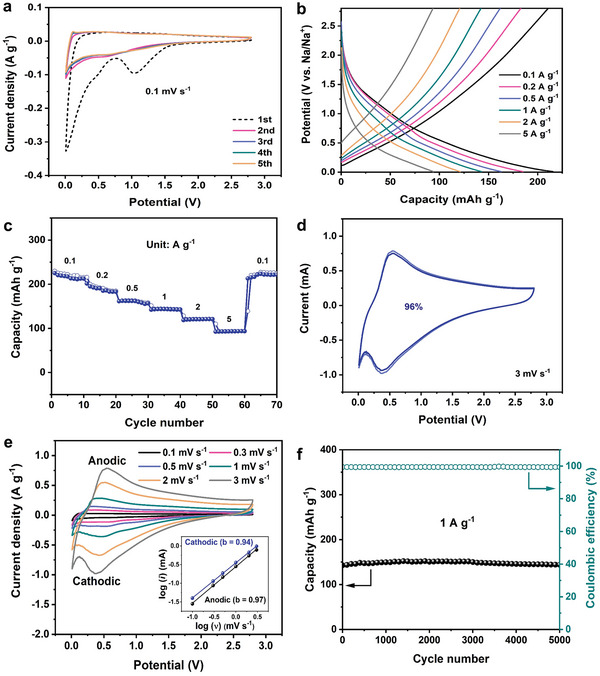
Electrochemical performances of MDC anode. a) CV curves during the 1st–5th cycles at a scan rate of 0.1 mV s^−1^. b) GCD profiles at different current densities. c) Rate performances. d) Capacitive contribution at a scan rate of 3 mV s^−1^. e) CV curves at scan rates from 0.1 to 3 mV s^−1^ (inset: plot of log (current) vs. log (scan rate) at anodic and cathodic peaks). f) Cycling stability at 1 A g^−1^.

The electrochemical properties of MDC anode were also evaluated in a potential range of 0.0–2.8 V (vs. Na/Na^+^). The CV curves at a scan rate of 0.1 mV s^−1^ are shown in **Figure** **5**a. The anodic peaks in the initial cycle of the CV curve originate from the irreversible reaction between the surface functional groups and sodium species, the decomposition of electrolytes, and the formation of a solid electrolyte interface (SEI) film.^[^ ^48^ ^]^ Besides, the shapes of the following CV curves suggest the reversibility of MDC anode, as well as the stable formation of the SEI film. In addition, the GCD profiles (Figure 5b) and rate capabilities (Figure 5c) support that a high‐capacity/high‐rate carbonaceous anode could be obtained via the simple one‐step pyrolysis of MAF‐6. MDC anode exhibits excellent rate capability, as demonstrated by the reversible specific capacities of 215, 185, 163, 143, 121, and 94 mAh g^−1^ at 0.1, 0.2, 0.5, 1, 2, and 5 A g^−1^, respectively, in the potential range of 0.0–2.8 V. When the current density is returned to 0.1 A g^−1^, the specific capacity is recovered to a value of 217 mAh g^−1^, proving the high reversibility of MDC anode. Figure 5d and Figure S16 (Supporting Information) support that MDC anode leads to the capacitive contribution of 96% at a scan rate of 3 mV s^−1^. Furthermore, the CV curves at various scan rates from 0.1 to 3 mV s^−1^ were presented to further explore the kinetics (Figure 5e). The obtained *b* values at the anodic and cathodic peaks, from the plot of log(*i*) versus log(*υ*), are 0.97 and 0.94, respectively, demonstrating that the capacitiveprocess is dominant. This noticeably high contribution of capacitance originates from the carbon doped with N via the pyrolysis of N‐containing compounds, MAF‐6. N species induced not only pseudocapacitance, but also the formation of defects that enhance the electrochemical activity.^[^ ^49^ ^]^ Moreover, the significantly high surface‐controlled capacitive contribution of MDC anode can be explained by the adsorption energy.^[^ ^50^, ^51^ ^]^ The adsorption property of Na is beneficially affected by N‐doped defects leading to capacitive dominant charge storage.^[^ ^52^ ^]^ Moreover, the specific capacity of MDC anode was well maintained over 5000 cycles at 1 A g^−1^, as demonstrated by the cycling stability results (Figure 5f). The capacity slowly increased in the front region of the cyclic test, attributed to improved wettability between the electrolyte and the electrode.^[^ ^3^ ^]^ Additionally, the electrochemically superior properties in capacity retention and rate capability are attributed to a capacitive dominant charge storage process that allows the fast kinetics of MDC anode. The high specific capacity, outstanding rate performance, and excellent cyclic stability imply that MDC can play as a competitive anode for SIHCs.

**Figure 6:**
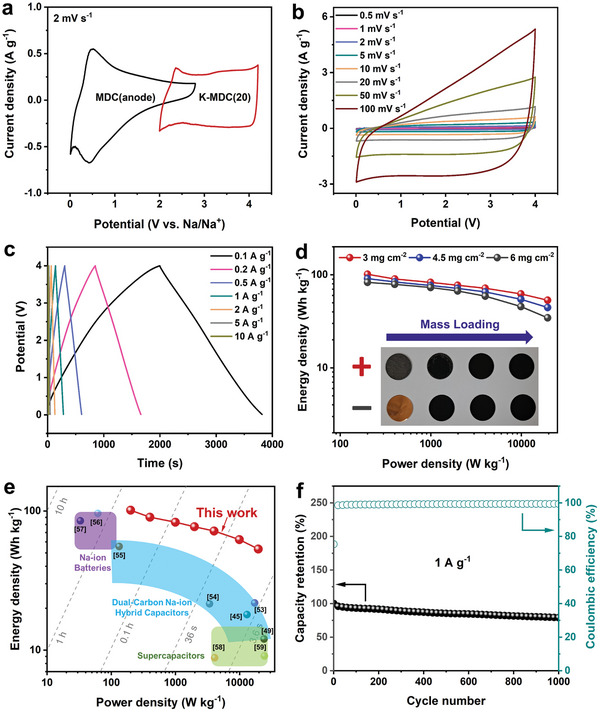
Electrochemical performances of MDC//K‐MDC SIHC full cells. a) CV curves of MDC anode and K‐MDC cathode at a scan rate of 2 mV s^−1^. b) CV curves of full cells at various scan rates (from 0.5 to 100 mV s^−1^). c) GCD profiles at different current densities. d) Ragone plot of MDC//K‐MDC full cells with the different mass loadings. e) Ragone plot compared with other reported dual‐carbon SIHCs, Na‐ion batteries, and supercapacitors. f) Cycling stability at 1 A g^−1^ over 1000 cycles.

Moreover, SIHCs were assembled using MDC anode and K‐MDC cathode (K‐MDC(20)), respectively, with different mass ratios. The prepared SIHCs were electrochemically evaluated in the potential range of 0.0–4.0 V based on the CV curves of the anode and cathode, as shown in **Figure** **6**a. The quasi‐rectangular shape of the CV curves of the full cells with the mass ratios of 1:2, 1:1, and 1:3 are shown in Figure 6b and Figure S17a,b (Supporting Information), respectively. The GCD curves for the three full cells in Figure 6c and Figure S17c,d (Supporting Information) are approximately linear. The optimized mass ratio of the anode to cathode is 1:2, which was determined using the Ragone plot of MDC//K‐MDC SIHC full cell obtained from the GCD profiles in Figure S18 (Supporting Information). The energy density and power density of the optimized full cell from the current density of 0.1–10 A g^−1^ are presented in Table S7 (Supporting Information). Figure S19 (Supporting Information) reveals the full‐cell capacities of 49 and 27 mAh g^−1^ at 0.1 and 10 A g^−1^, respectively. In addition, Figure 6d shows the energy densities of full cells with different mass loadings ranging from 3 to 6 mg cm^−2^. This demonstrates that the energy densities were well maintained even at a high‐mass loading. Figure 6e and Table S8 (Supporting Information) show the Ragone plots and obtained energy/power densities of the optimized MDC//K‐MDC SIHC, previous other dual‐carbon SIHCs, sodium‐ion batteries, and supercapacitors. MDC//K‐MDC(20) SIHC is quite competitive against other reported works, such as HAT‐CNF‐850//STC‐16,^[^ ^45^ ^]^ HAT550@ZTC//STC‐16,^[^ ^49^ ^]^ graphite//AC,^[^ ^53^ ^]^ PI/rGO‐rGO//rGO,^[^ ^54^ ^]^ GCNF//AC,^[^ ^55^ ^]^ Na_0.8_Ni_0.4_Ti_0.6_O_2_//Na_0.8_Ni_0.4_Ti_0.6_O_2_,^[^ ^56^ ^]^ Na_0.66_[Li_0.22_Ti_0.78_]O_2_//Na_3_V_2_(PO_4_)_3_/C,^[^ ^57^ ^]^ LS AC//LS AC,^[^ ^58^ ^]^ and ZBTC_0.8_‐900// ZBTC_0.8_‐900.^[^ ^59^ ^]^ Importantly, the energy density of 53.2 Wh kg^−1^ was delivered even at an ultrafast chargeable power density of 20000 W kg^−1^ outperforming that of a battery by ≈100‐folds. Additionally, Figure 6f shows the cycling stability test of MDC//K‐MDC SIHC at 1 A g^−1^ and it demonstrates that ≈80% of capacity was retained during 1000 cycles. On the other hand, we expect that the cycle stability of dual‐carbon capacitors could be further improved by using conductive carbon shell frameworks, such as reduced graphene shells on MOFs.^[^ ^30^ ^]^

## 3 Conclusion

In summary, we utilized MAF‐6s to synthesize 3D porous oxygen‐doped and nitrogen‐doped graphitic carbons as cathode and anode materials for high‐performance dual‐carbon SIHCs. First, the carbonization of MAF‐6s after loading urea in a suitable quantity was made to synthesize MDC structures. Then, K‐MDC structures with micropores and mesopores were selectively synthesized as cathode materials by controlling the N‐content of MDCs in the presence of KOH. The optimized K‐MDC(20), called as K‐MDC, exhibited a record‐high surface area (5214 m^2^ g^−1^), which is ≈four‐fold higher than that of pristine MAF‐6 and is the highest one among carbonaceous cathode materials, as well as rich oxygen‐doped sites allowing high capacity for electrolyte anions (ClO_4_^−^). Also, it showed rich interconnected open mesopores allowing fast ion transport and high capacity retention over 5000 charge/discharge cycles. Moreover, N‐containing MAF‐6s were successfully converted into 3D porous N‐doped graphitic carbons (MDC anode materials). They were demonstrated to allow excellent capacity retention over 5000 cycles. Furthermore, assembling K‐MDC cathode and MDC anode into a dual‐carbon full‐cell configuration showed remarkable performances in a SIHC, as exhibited by a high energy density exceeding those of sodium‐ion batteries and supercapacitors. Besides, high energy densities were well maintained even at different mass loadings ranging from 3 to 6 mg cm^−2^. Additionally, it achieved a fast‐chargeable ultrahigh power density (20000 W kg^−1^) and robust stability over 1000 cycles. The superior properties of MDC//K‐MDC SIHCs originate from the kinetics and charge storage mechanism match between the 3D porous oxygen‐doped graphitic carbon cathode and nitrogen‐doped graphitic carbon anode materials derived from the same precursor, MAF‐6. Consequently, this work not only suggests a strategy to develop 3D porous carbons, but also provides a way to fabricate high‐performance dual‐carbon SIHCs affording high energy density and power densities, as well as robust cycle stability.

## 4 Experimental Section

### Chemicals

All chemicals used in this study were procured from local vendors and used without further purification. Details of the materials are described in Supporting Information.

### Preparation of MAF‐6s

First, Zn(OH)_2_ (1.988 g, 20 mmol) was dissolved in NH_4_OH (25%, 400 mL) to prepare solution A. Simultaneously, 3.8 g of 2‐ethylimidazole (40 mmol) was dissolved in a mixture of ethanol (300 mL) and cyclohexane (20 mL) to prepare solution B. Then, solution A was added to solution B, and the mixture was stirred for 30 min. After that, the precipitate was filtered from the solution, and the obtained solid was washed with ethanol. The obtained white product was dried in a vacuum oven to obtain MAF‐6 powder. Urea‐loaded MAF‐6 samples were prepared by wet impregnation. MAF‐6 (1 g) was added to 20 mL of ethanolic solution of urea (0.2 or 0.5 g), mixed well for 30 min under sonication, and stirred for 12 h. The liquid part of the mixture was removed by rotary evaporation. The urea‐loaded MAF‐6 samples were named MAF‐6_Ur*x*, where *x* indicates the percentage of the loaded urea against the weight of MAF‐6. The samples were stored for further use after drying at 80 °C.

### Preparation of MDCs

The prepared MAF‐6s, with or without urea loading, were pyrolyzed to obtain MAF‐6‐derived carbons (called MDC(*x*)). MAF‐6 (or MAF‐6‐Ur20, MAF‐6‐Ur50) sample (1 g) was loaded onto a tubular furnace and heated under Ar flow to 800°C and maintained for 1 h for pyrolysis. After cooling down the furnace to room temperature, the samples were recovered and soaked in a concentrated HCl solution to remove the residual Zn species. Then, the slurry was filtered, and the solid was recovered by washing it several times with deionized water. Finally, the product was dried for 12 h in a vacuum oven at 60 °C and stored for the second pyrolysis with the KOH activator. For MDC anode material, MAF‐6 was similarly pyrolyzed, but MAF‐6 without urea was carbonized in one step at 900 °C for 2 h.

### Preparation of K‐MDCs

KOH and MDC(*x*) that was obtained in the first pyrolysis were mixed together (2:1, wt:wt) in deionized water with stirring. After 2 h of mixing, the slurry was separated using a rotary evaporator and further dried in a vacuum oven at 60 °C. The dried solid was put into the tubular furnace and carbonized at 800 °C for 1 h under Ar flow. The resultant product was then soaked in dilute HCl (10%) for 2 h to remove residual KOH and Zn. Finally, the solid product was recovered from the solution with filtration and dried at 60 °C in a vacuum oven. The final product was named K‐MDC(*x*), where *x* indicates the weight% of urea against MAF‐6 in the mixtures for the first pyrolysis.

### Materials Characterization

The crystal structures of MAF‐6 and carbons were analyzed using X‐ray diffraction (XRD, Rigaku, SmartLab X‐ray diffractometer) with Cu–K*α*1 radiation at 1200 W (40 kV, 30 mA). Transmission electron microscopy (TEM, JEOL, JEM‐ARM200F) and scanning electron microscopy (SEM, Hitachi, SU8230) analyses were performed to examine the morphologies of the samples. The surface properties were characterized by X‐ray photoelectron spectroscopy (XPS, Thermo Scientific, Thermo VG Scientific K‐alpha spectrometer) equipped with Al–K*α* X‐ray monochromator (incident photon energy: 1486.6 eV). The textural properties of samples were analyzed using nitrogen adsorption–desorption isotherms measured at 77 K with Quadrasorp (Quantachrome Instruments). The specific surface area and the pore size distribution (PSD) of samples were calculated using Brunauer–Emmett–Teller (BET) equation and the non‐local density functional theory (NLDFT) method, respectively. Micro‐ and total‐pore volumes of the samples were calculated using Dubinin–Radushkevich method and the adsorbed quantity of N_2_, respectively.

### Evaluation of SIHCs

To prepare the working electrode of the cathode, K‐MDCs, carbon black (Super‐P), and polyvinylidene fluoride (PVDF) were homogeneously mixed with a mass ratio of 8:1:1 in *N*‐methyl‐2‐pyrrolidinone (NMP). Then, the slurry was coated on carbon paper and dried at 80 °C in a vacuum oven. To fabricate the working electrode of an anode, MDC(anode), carbon black (Super‐P), and polyvinylidene fluoride (PVDF) were mixed at a mass ratio of 7:2:1 in *N*‐methyl‐2‐pyrrolidinone (NMP) and coated on copper foil, followed by drying at 80°C in a vacuum oven. The dried cathode (2–4 mg cm^−2^) and anode (1–2 mg cm^−2^) electrodes were punched out and 2032‐type coin cells were assembled in an Ar‐filled glove box with a sodium metal, a glass fiber separator, and 1 m NaClO_4_ in EC/DEC (1:1 v/v) electrolyte. CV and GCD tests were employed on a VSP potentiostat equipment (Bio‐logic) and a battery cycler (Wonatech, WBCS‐3000). CV and GCD measurements of the anode and cathode were performed at operating voltages of 0–2.8 and 2–4.2 V, respectively. The SIHC full cell was assembled in an Ar‐filled glove box using K‐MDC cathode and pre‐sodiated MDC electrodes with the mass loadings in 3–6 mg cm^−2^ for active anode and cathode materials. The CV and GCD profiles of full cells were measured at the operating voltage of 0–4 V. The power density (P, W kg^−1^) and energy density (E, Wh kg^−1^) were calculated using the following equations of

(3) $$V=\left(V_{\max}+V_{\min}\right)/2$$

(4) P=V×i/m

(5) E=P×t/3600

where *V*_max_ and *V*_min_ are the maximum and minimum voltages, respectively, *i* is the applied current, *m* is the total mass (g) of the active materials in both anode and cathode, and *t* is the discharge time (s).

## Conflict of Interest

The authors declare no conflict of interest.

## Supporting information

Supporting Information

## Data Availability

The data that support the findings of this study are available from the corresponding author upon reasonable request.
