# Supplementary material for: 3D Porous Oxygen‐Doped and Nitrogen‐Doped Graphitic Carbons Derived from Metal Azolate Frameworks as Cathode and Anode Materials for High‐Performance Dual‐Carbon Sodium‐Ion Hybrid Capacitors
Source: Adv Sci (Weinh). 2023 Jun 16;10(24):2301160. doi: 10.1002/advs.202301160 (PMC10460885; doi:10.1002/advs.202301160)
Supplement: Supplementary file 1 — Supporting Information [file ADVS-10-2301160-s001.pdf]

ADVANCED SCIENCE
Open Access

Supporting Information

for *Adv. Sci.*, DOI 10.1002/advs.202301160

3D Porous Oxygen-Doped and Nitrogen-Doped Graphitic Carbons Derived from Metal Azolate Frameworks as Cathode and Anode Materials for High-Performance Dual-Carbon Sodium-Ion Hybrid Capacitors

*Yong Min Jung, Jong Hui Choi, Dong Won Kim and Jeung Ku Kang**

Copyright WILEY-VCH Verlag GmbH & Co. KGaA, 69469 Weinheim, Germany, 2023.

Supporting Information

3-dimensional porous oxygen-doped and nitrogen-doped graphitic carbons derived from metal azolate frameworks as cathode and anode materials for high-performance dual-carbon sodium-ion hybrid capacitors

*Yong Min Jung, Jong Hui Choi, Dong Won Kim, Jeung Ku Kang**

Y. M. Jung, J. H. Choi, D. W. Kim, Prof. J. K. Kang
Department of Materials Science and Engineering
NanoCentury Institute
Korea Advanced Institute of Science and Technology (KAIST)
291 Daehak-ro, Yuseong-gu, Daejeon 34141, Republic of Korea
*Email: jeungku@kaist.ac.kr

Section S1. Chemicals for material synthesis

2-Ethylimidazole ($C_5H_8N_2$, 98%), urea (CH_4N_2O, 99%), potassium hydroxide (KOH, 85%), and cyclohexane (C_6H_{12}, 99%) were purchased from Sigma-Aldrich. Zinc hydroxide ($Zn(OH)_2$, 98%), hydrochloric acid (HCl, 35-37%) and ammonium hydroxide (NH_4OH, 25-30%) were provided by DUKSAN science. Ethanol (C_2H_5OH, 95%) was provided from SAMCHUN CHEMICALS. All the purchased chemicals were used without further purification.

Section S2. Supporting figures and tables

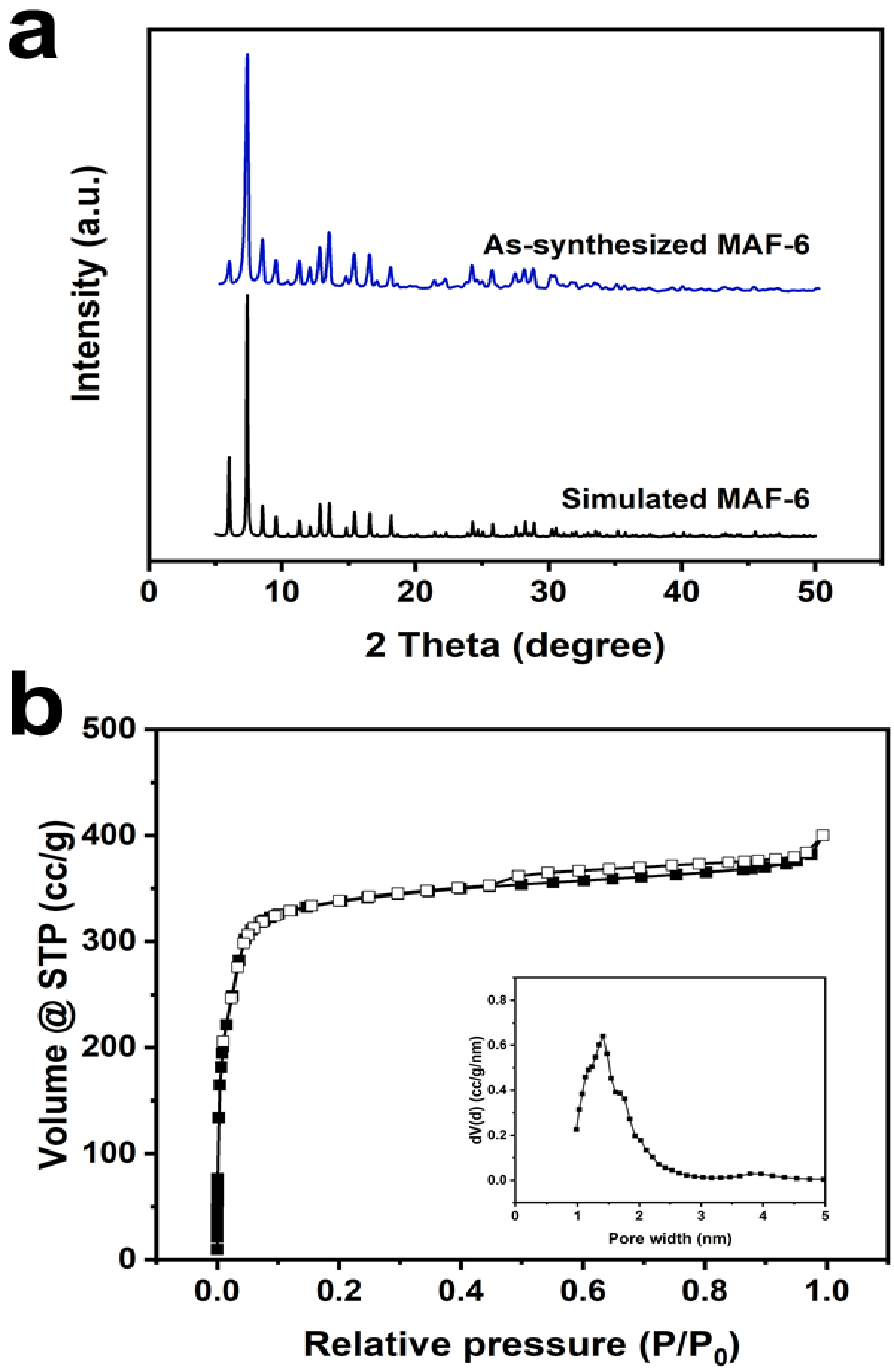

Figure S1. Structural characterizations of MAF-6. (a) X-ray diffraction (XRD) patterns and (b) N_2 adsorption-desorption isotherms (inset: DFT pore size distributions) of MAF-6.

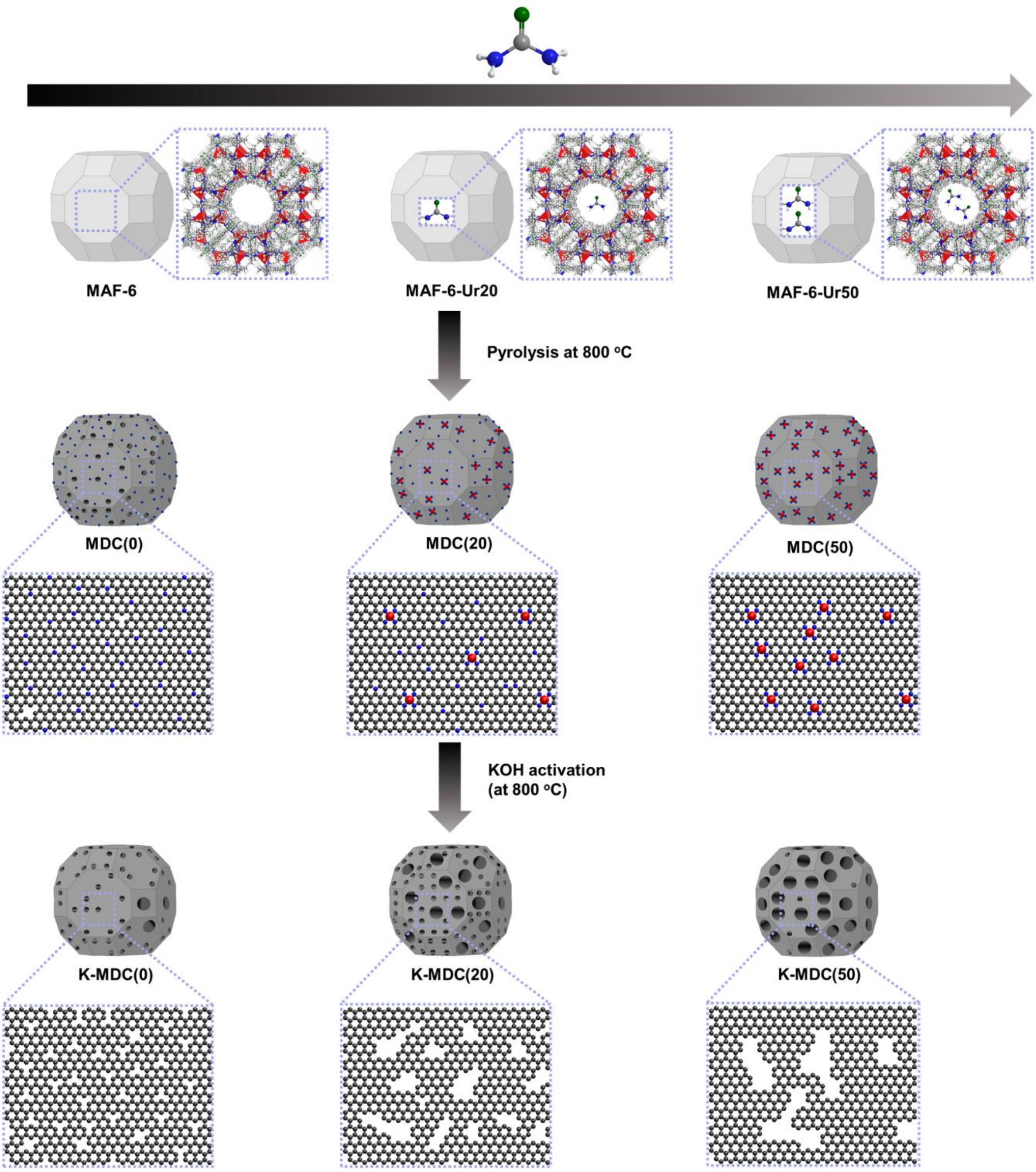

Figure S2. Schematic illustration of the synthetic process for cathode materials, K-MDCs.

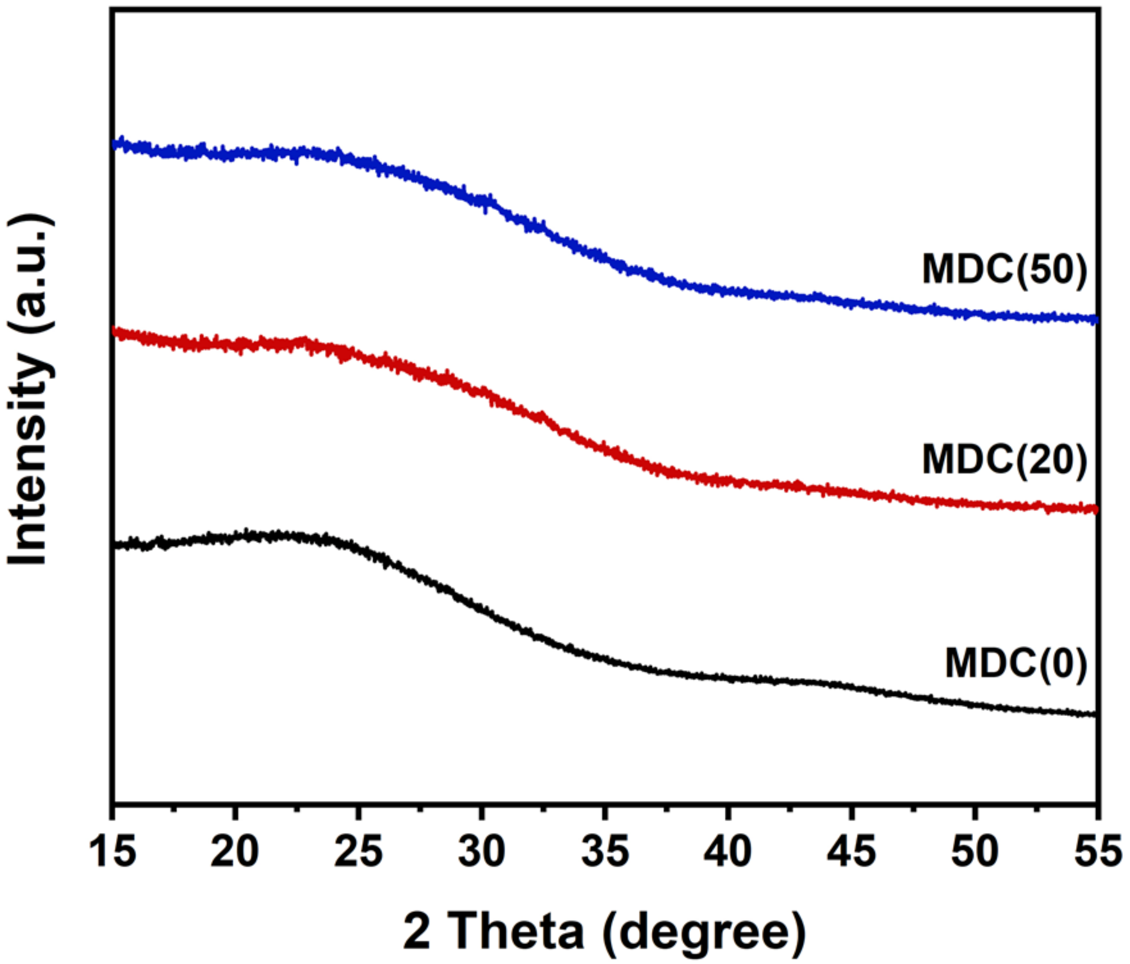

Figure S3. XRD patterns of MDCs.

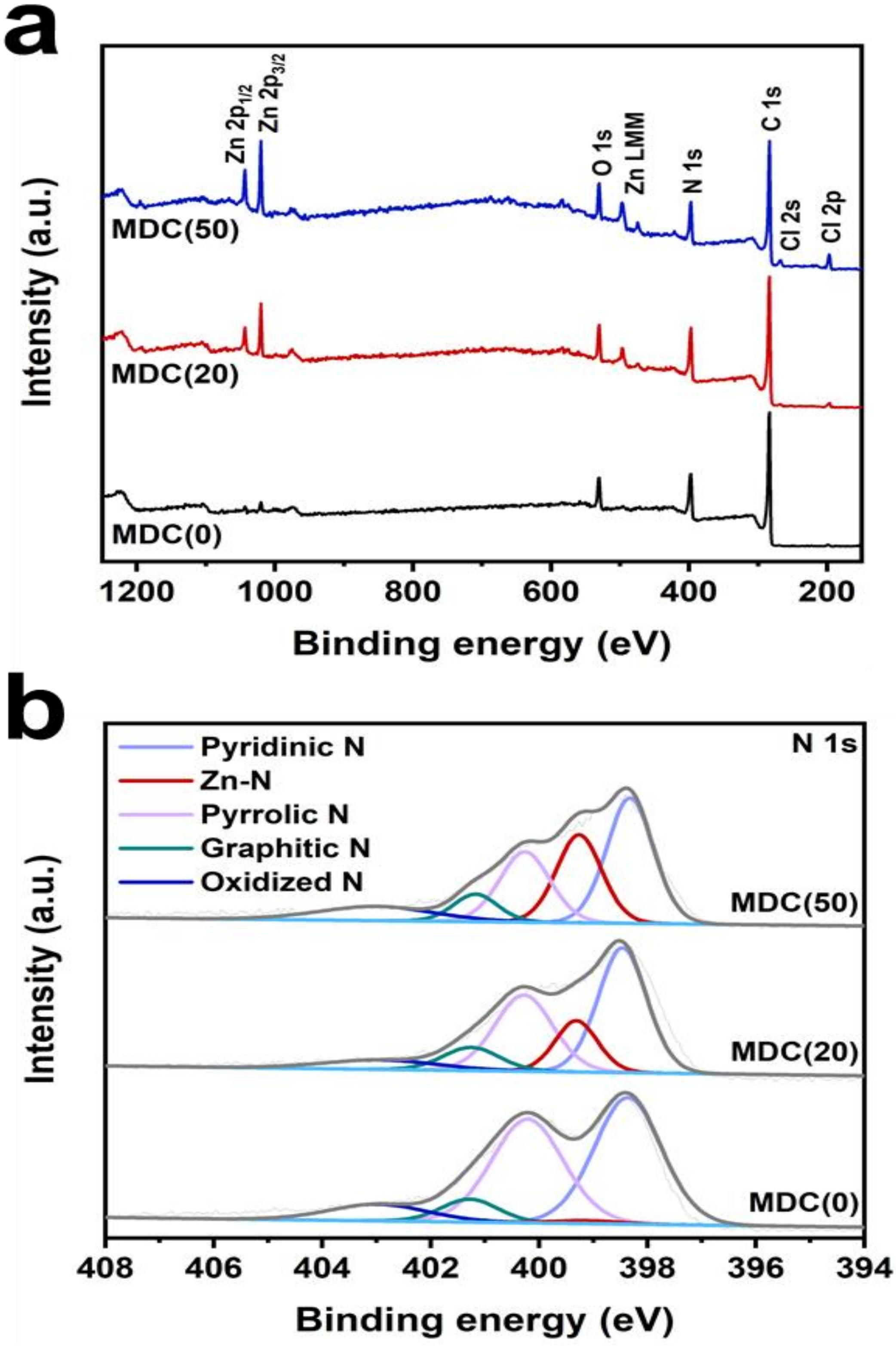

Figure S4. X-ray photoelectron spectroscopy (XPS) spectra. (a) Survey spectra and (b) High-resolution N 1s spectra of MDCs.

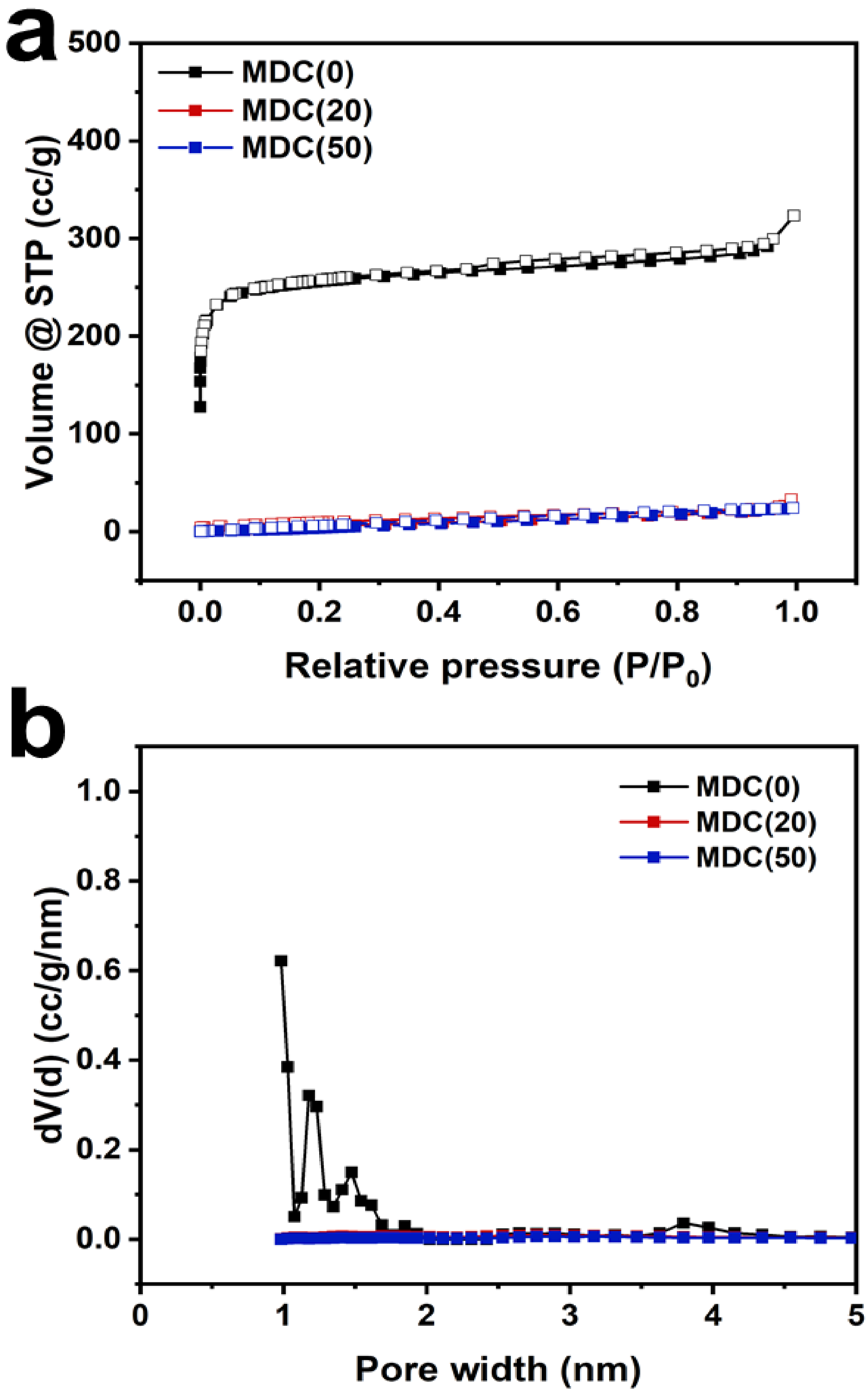

Figure S5. Pore volume and size analyses. (a) N_2 adsorption-desorption isotherms and (b) corresponding pore size distributions of MDCs.

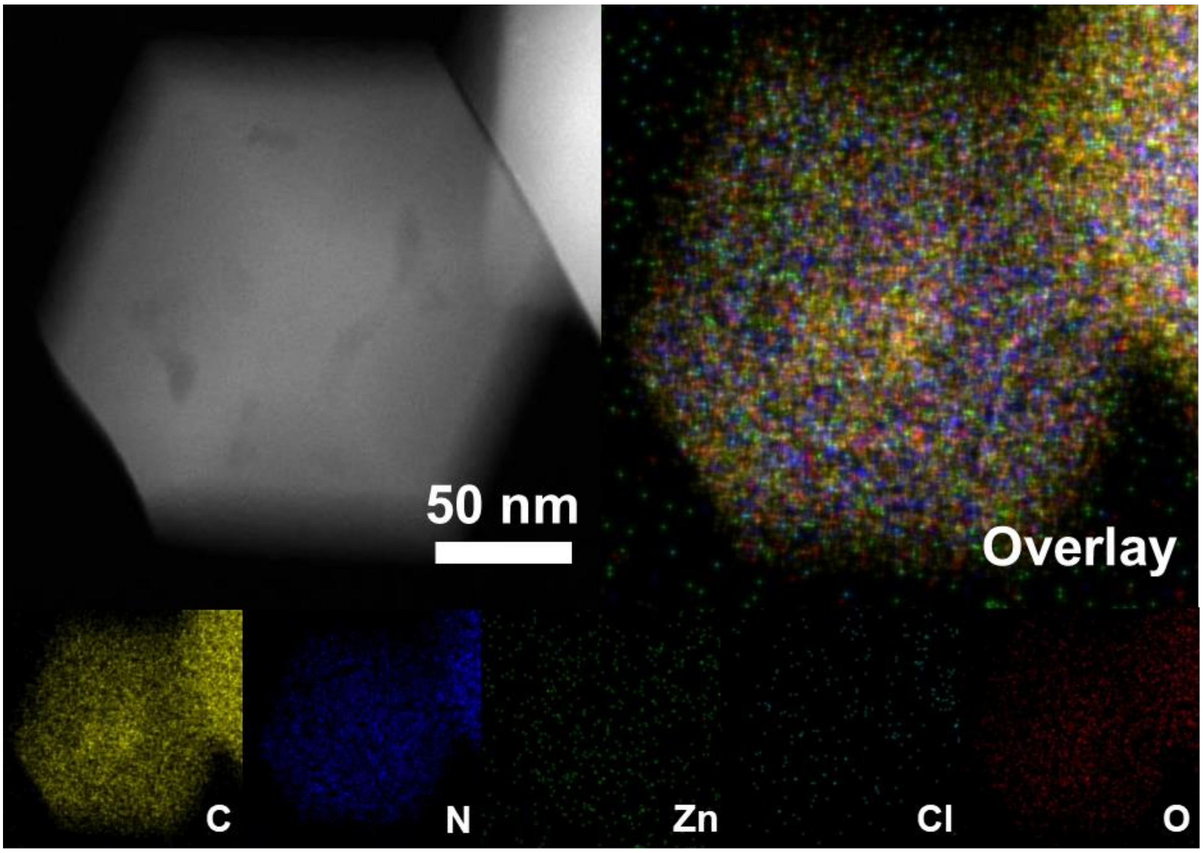

Figure S6. High-angle annular dark-field (HAADF)-scanning transmission electron microscopy (STEM) and energy dispersive spectroscopy (EDS)-mapping images of MDC(20).

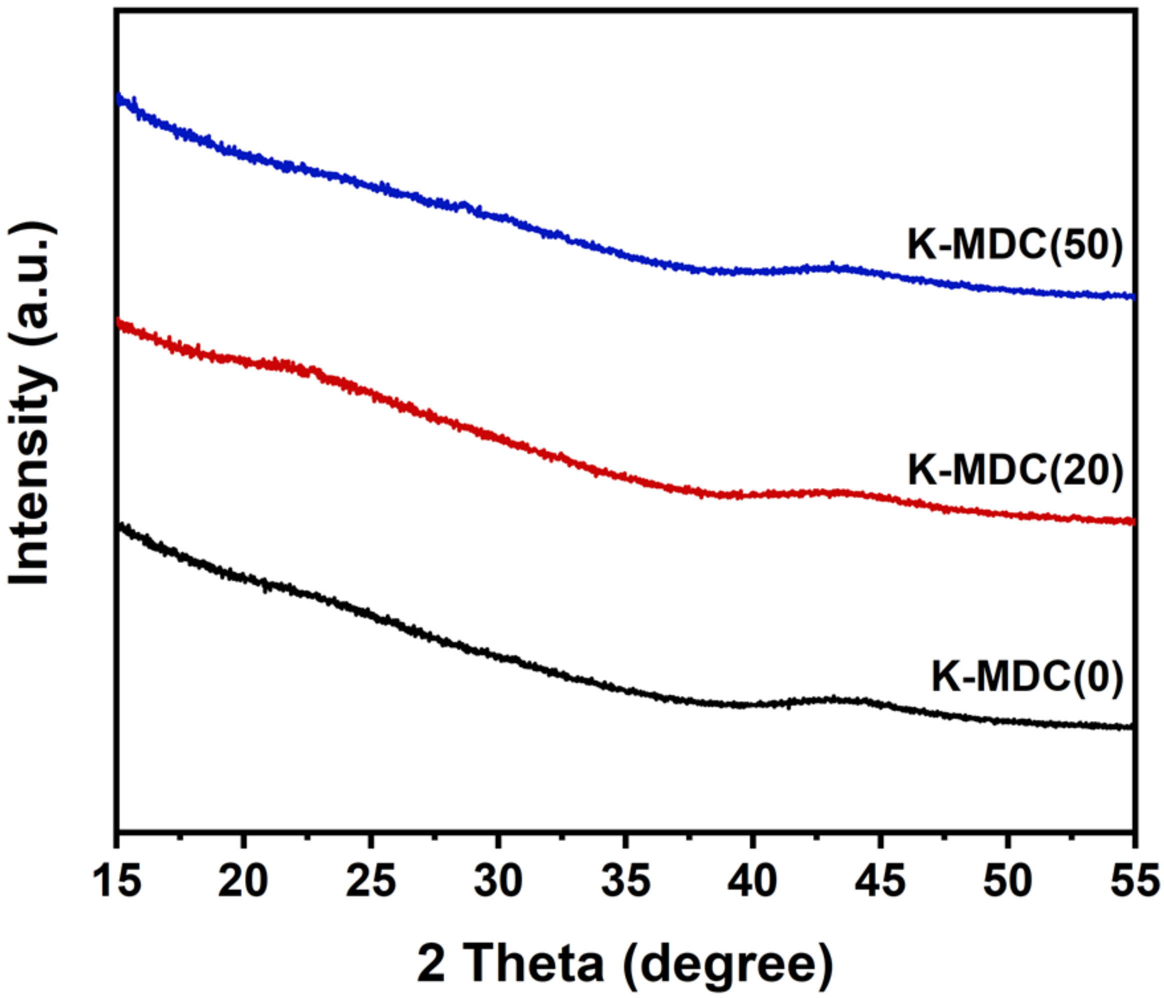

Figure S7. XRD patterns of K-MDCs.

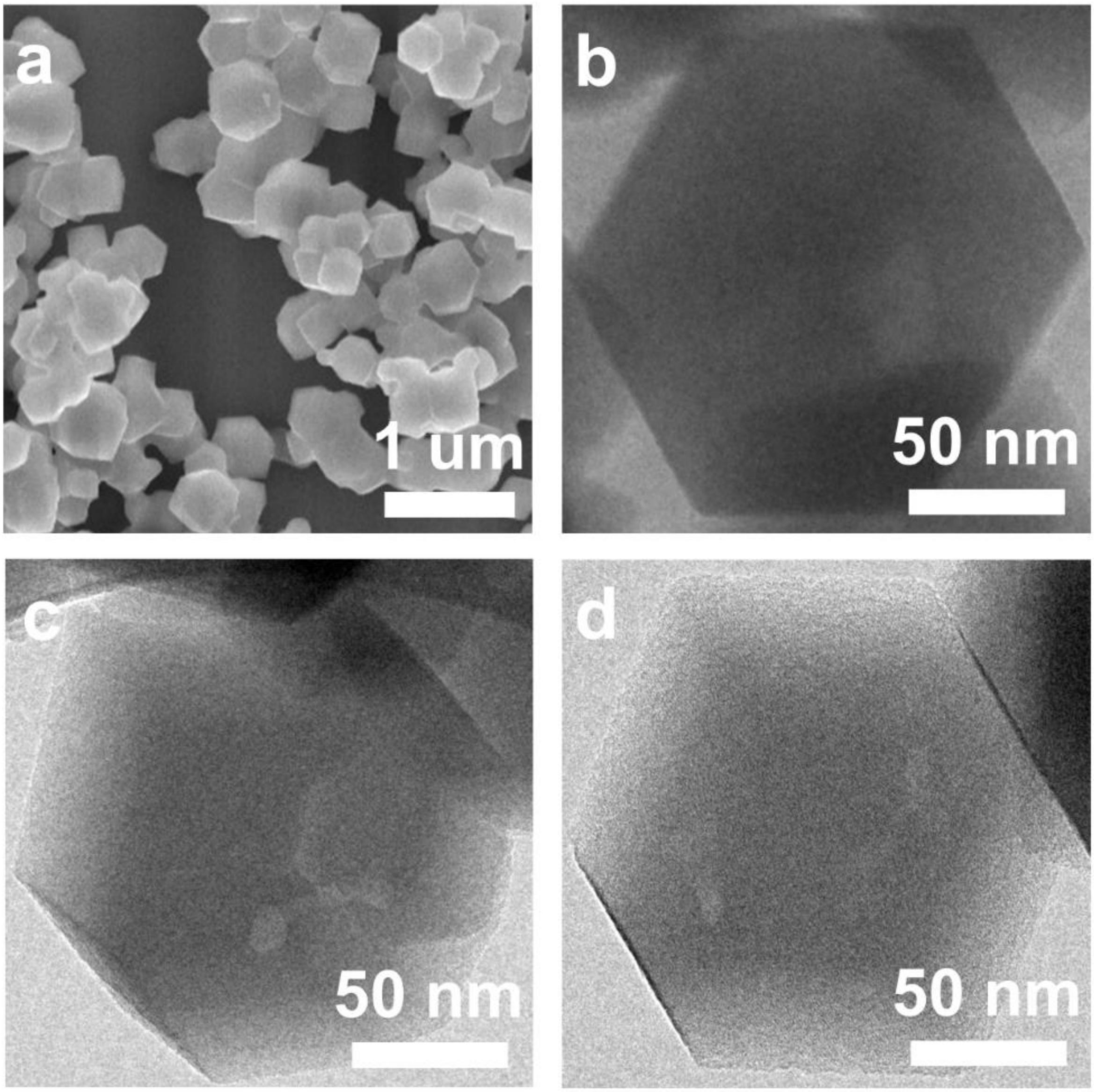

Figure S8. Scanning electron microscope (SEM) and transmission electron microscopy (TEM) image. (a) SEM image of MAF-6. TEM images of (b) MAF-6, (c) MDC(20), and (d) K-MDC(20).

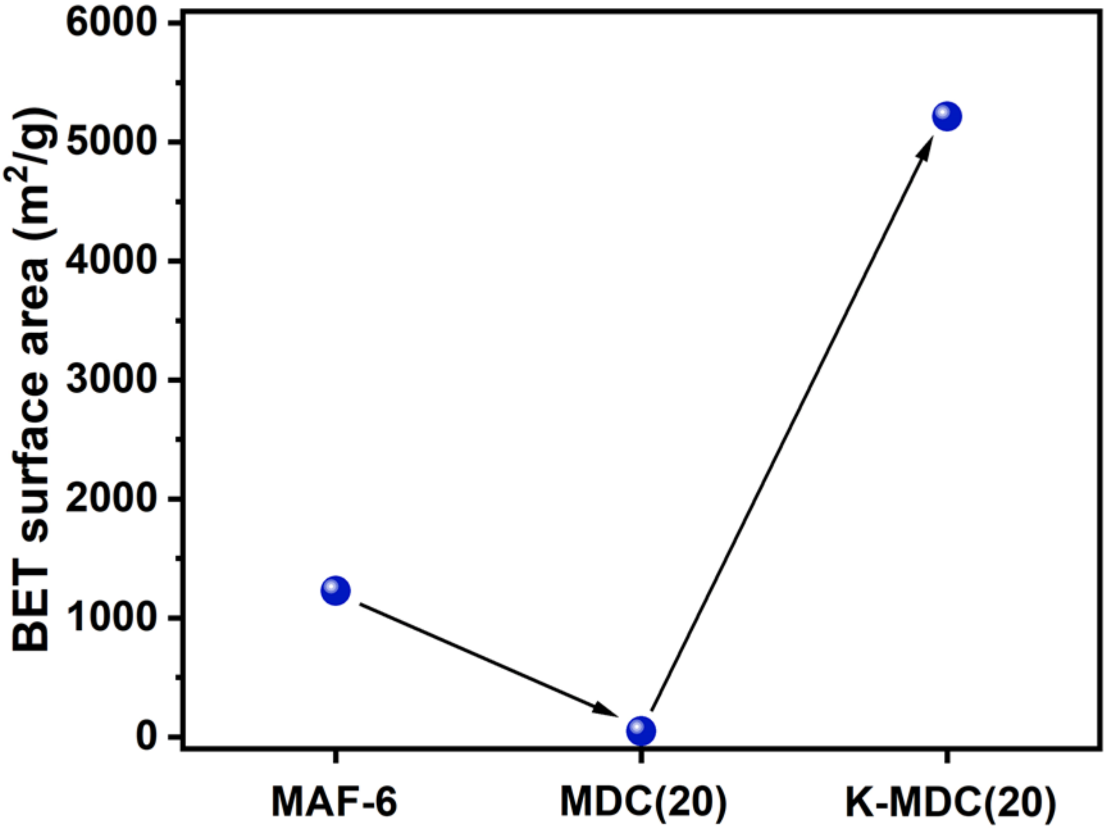

Figure S9. Change of BET (Brunauer, Emmett and Telle) surface area from MAF-6 to K-MDC(20).

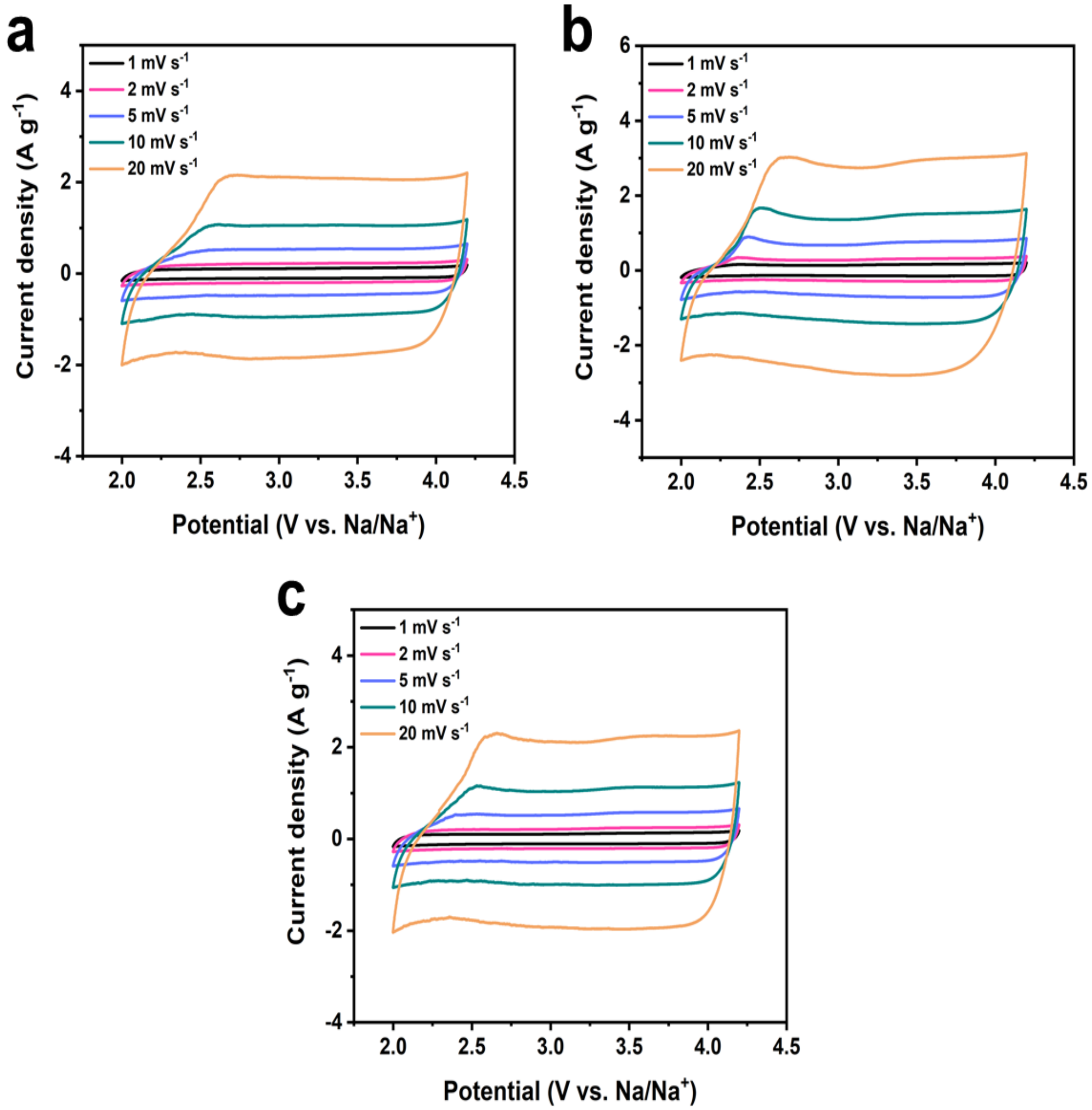

Figure S10. Cyclic voltammetry (CV) characteristics of cathode materials. CV curves of (a) K-MDC(0), (b) K-MDC(20), and (c) K-MDC(50) at various scan rates (from 1 mV s^{-1} to 20 mV s^{-1}).

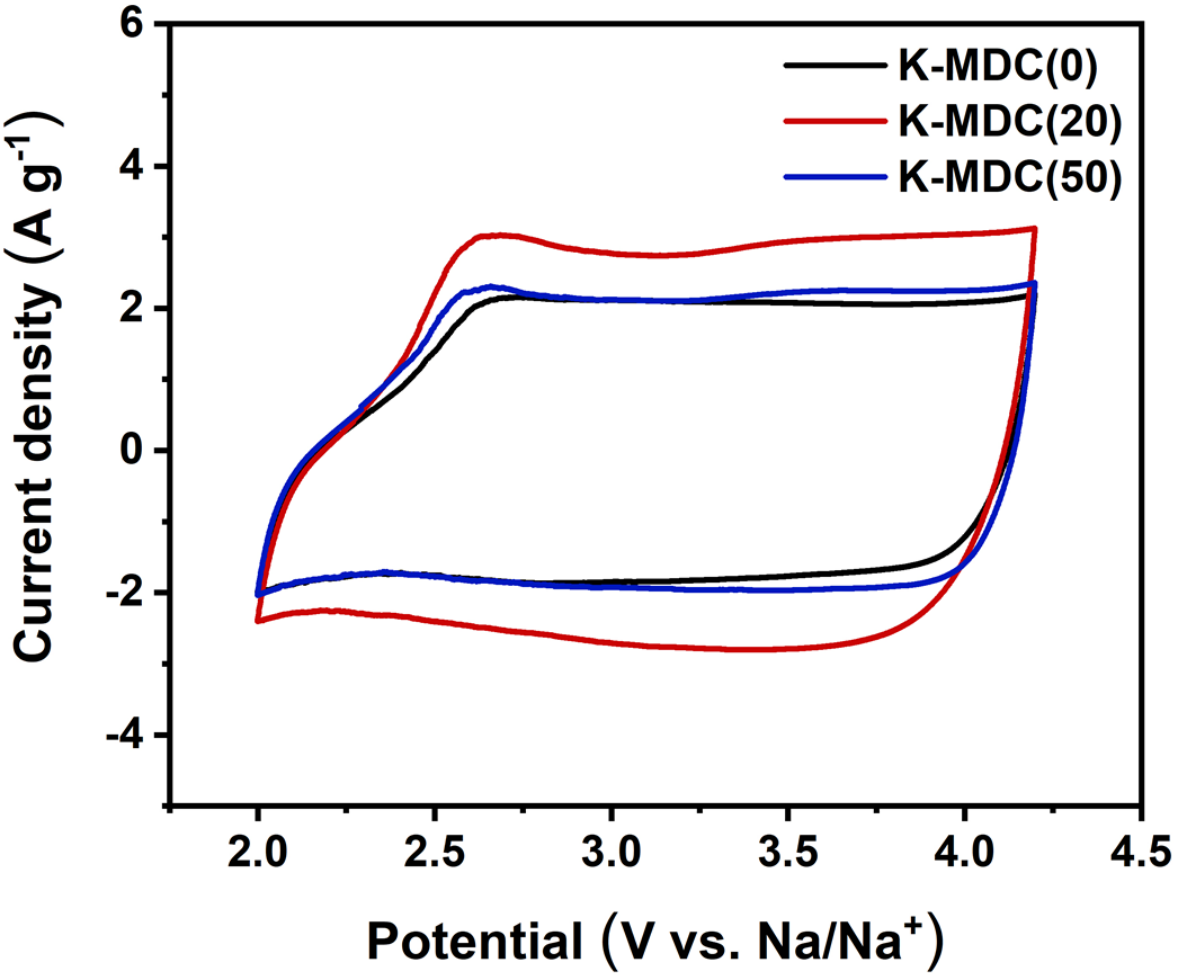

Figure S11. CV curves of K-MDCs at 20 mV s^{-1}.

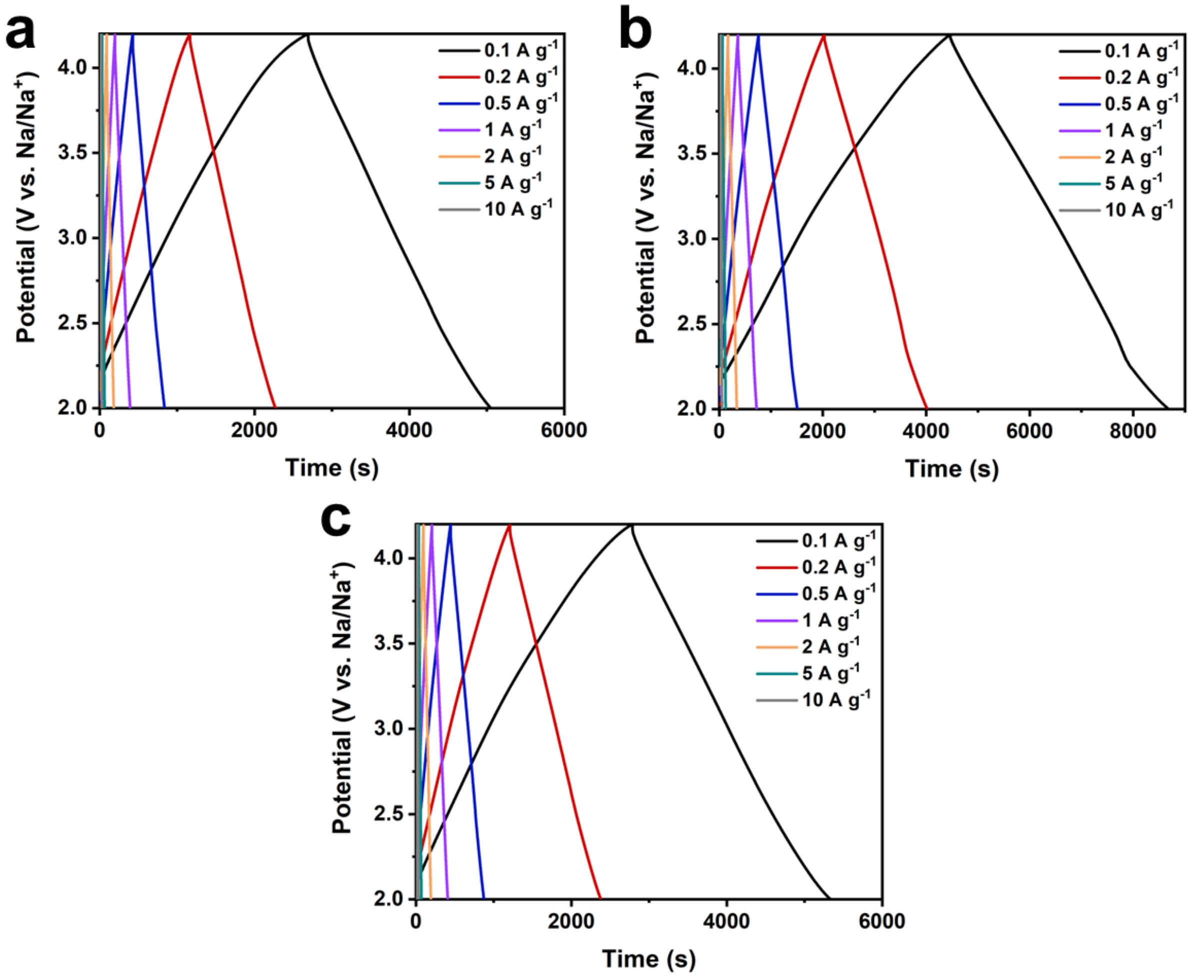

Figure S12. Galvanostatic charge/discharge (GCD) characteristics of cathode materials. GCD profiles of (a) K-MDC(0), (b) K-MDC(20), and (c) K-MDC(50) at different current densities.

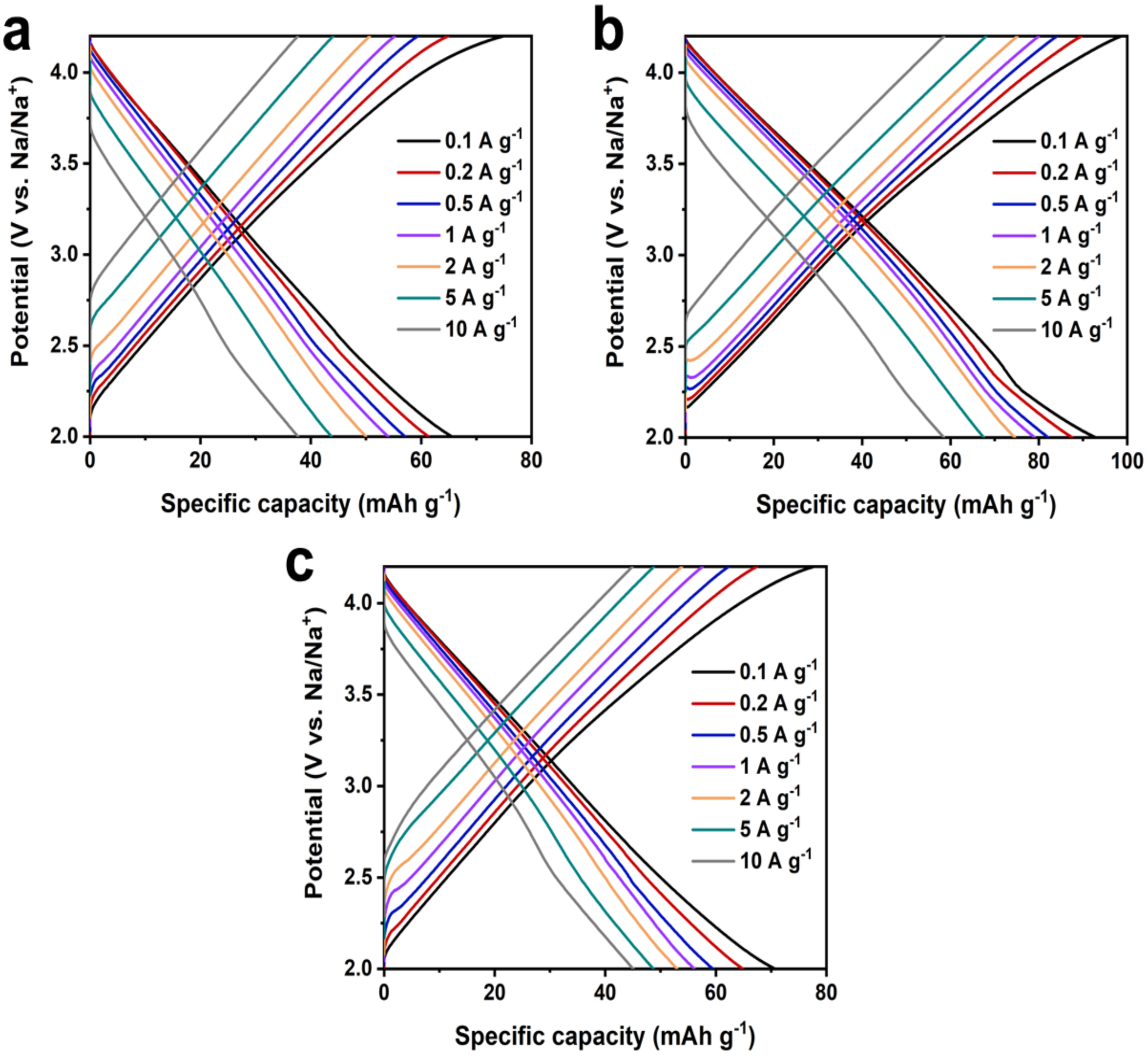

Figure S13. GCD characteristics of cathode materials. GCD profiles of (a) K-MDC(0), (b) K-MDC(20), and (c) K-MDC(50) at different current densities.

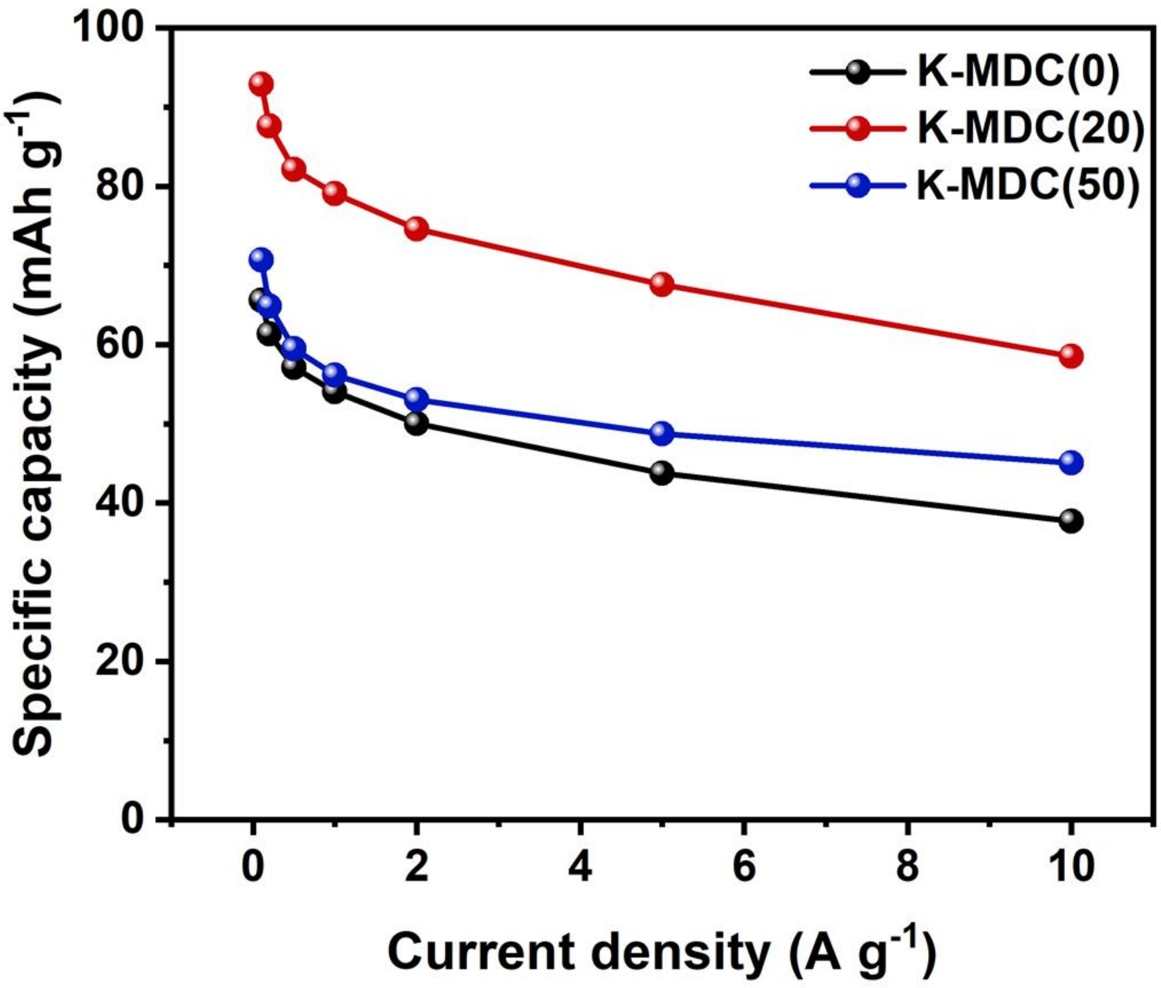

Figure S14. Specific capacities of K-MDCs at different current densities.

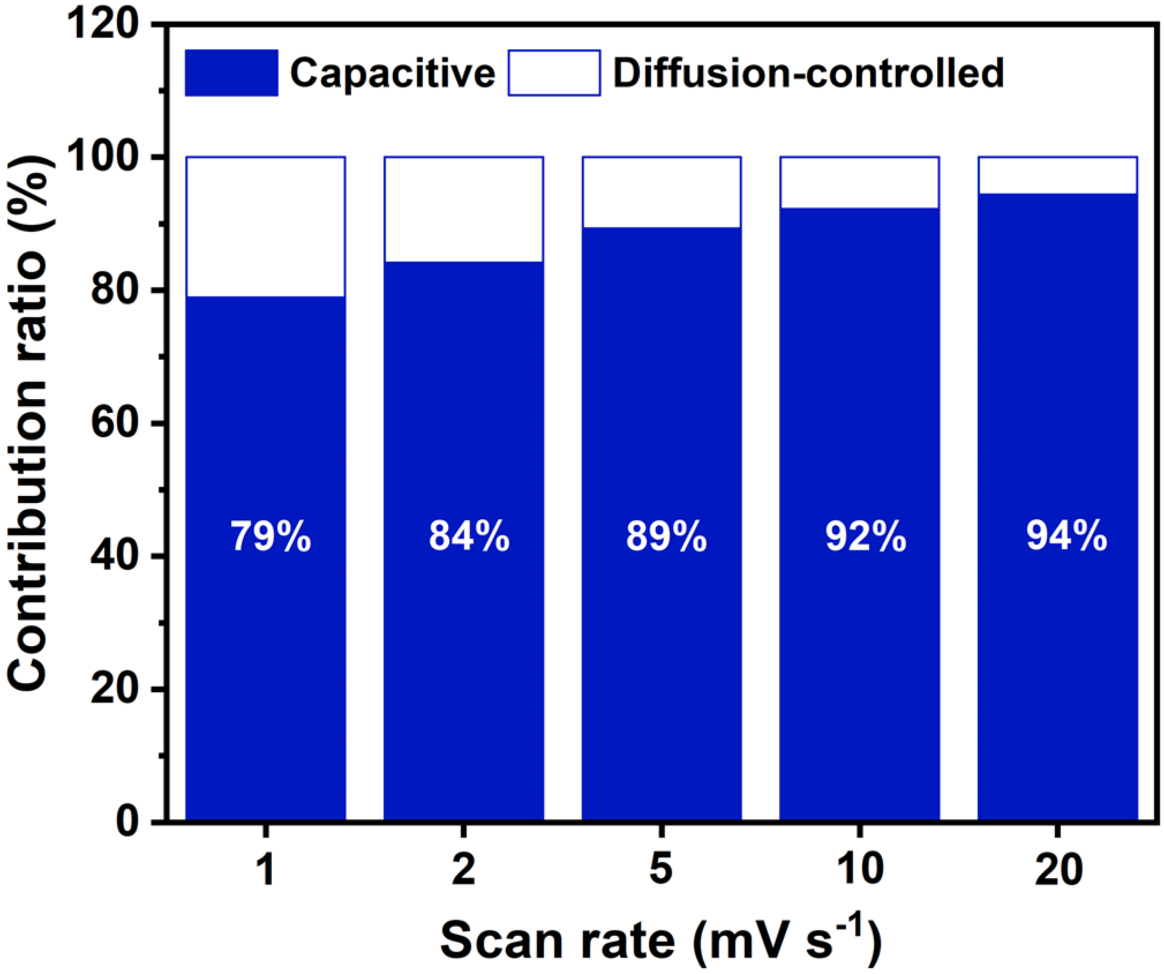

Figure S15. Contribution ratio of K-MDC(20) for capacitive and diffusion-controlled behavior at various scan rates.

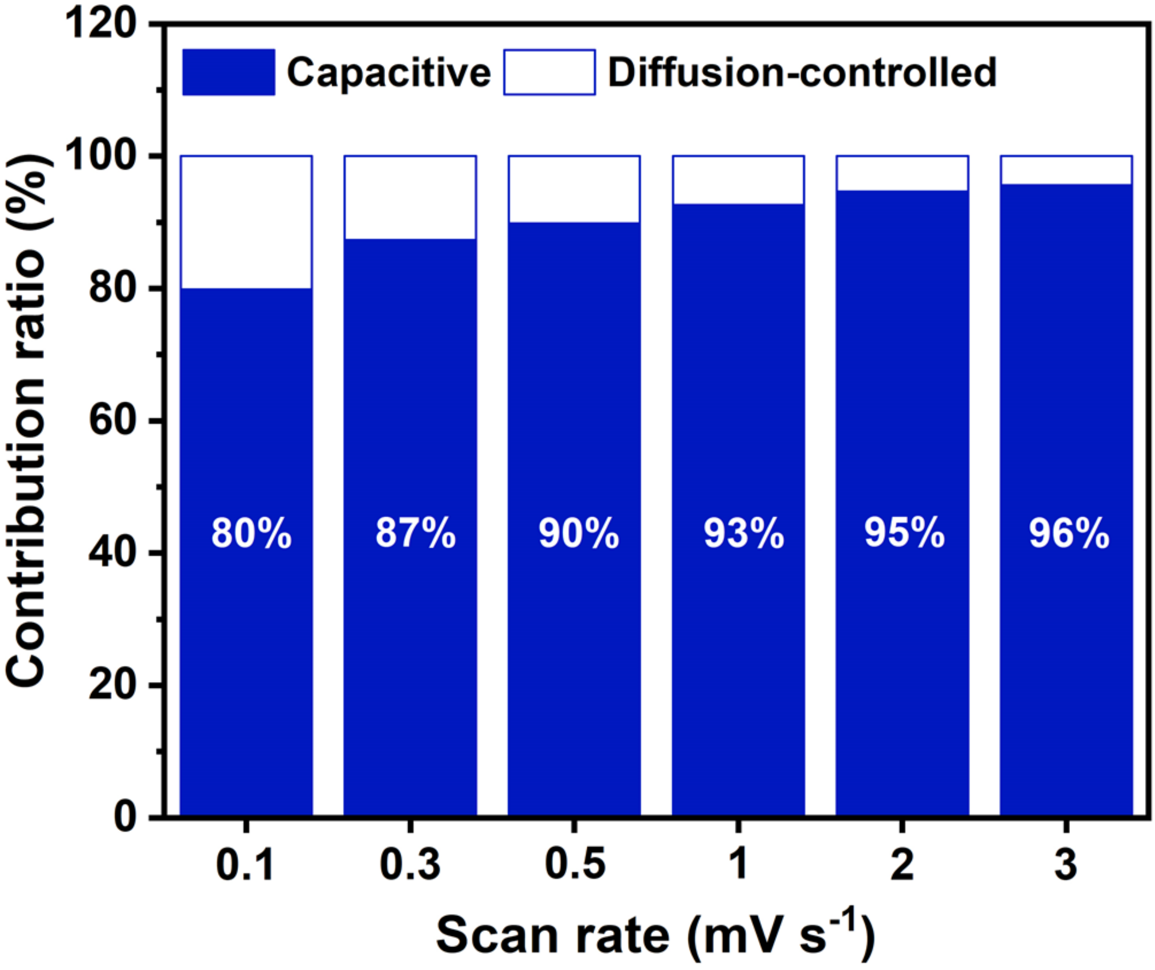

Figure S16. Contribution ratio of MDC anode for capacitive and diffusion-controlled behaviors at various scan rates.

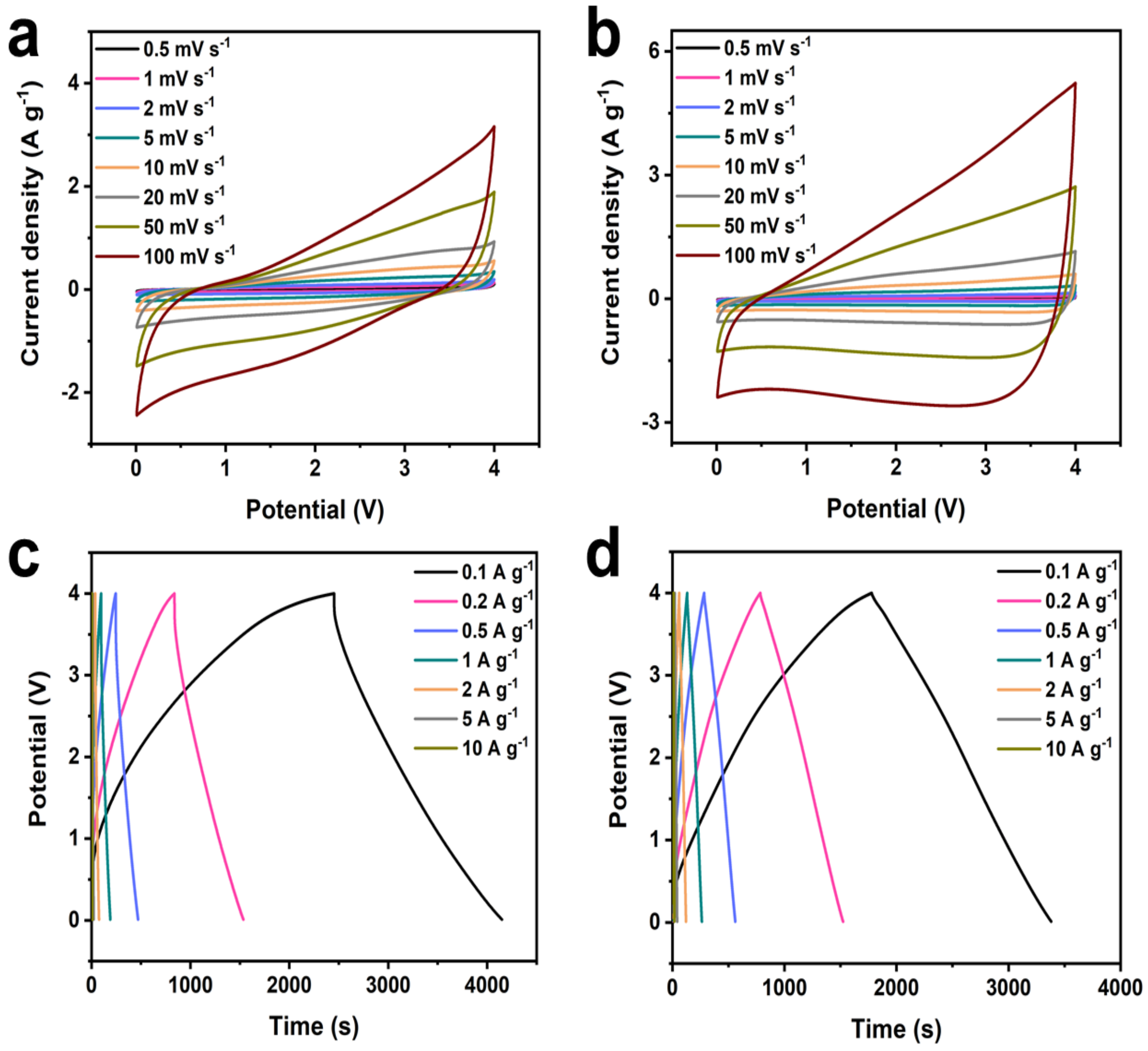

Figure S17. CV and GCD characteristics of MDC//K-MDC full cells. CV curves (a and b) and GCD curves (c and d) of MDC//K-MDC full cell with the different mass ratios of anode to cathode, where (a and c) anode to cathode is 1:1 and (b and d) anode to cathode is 1:3.

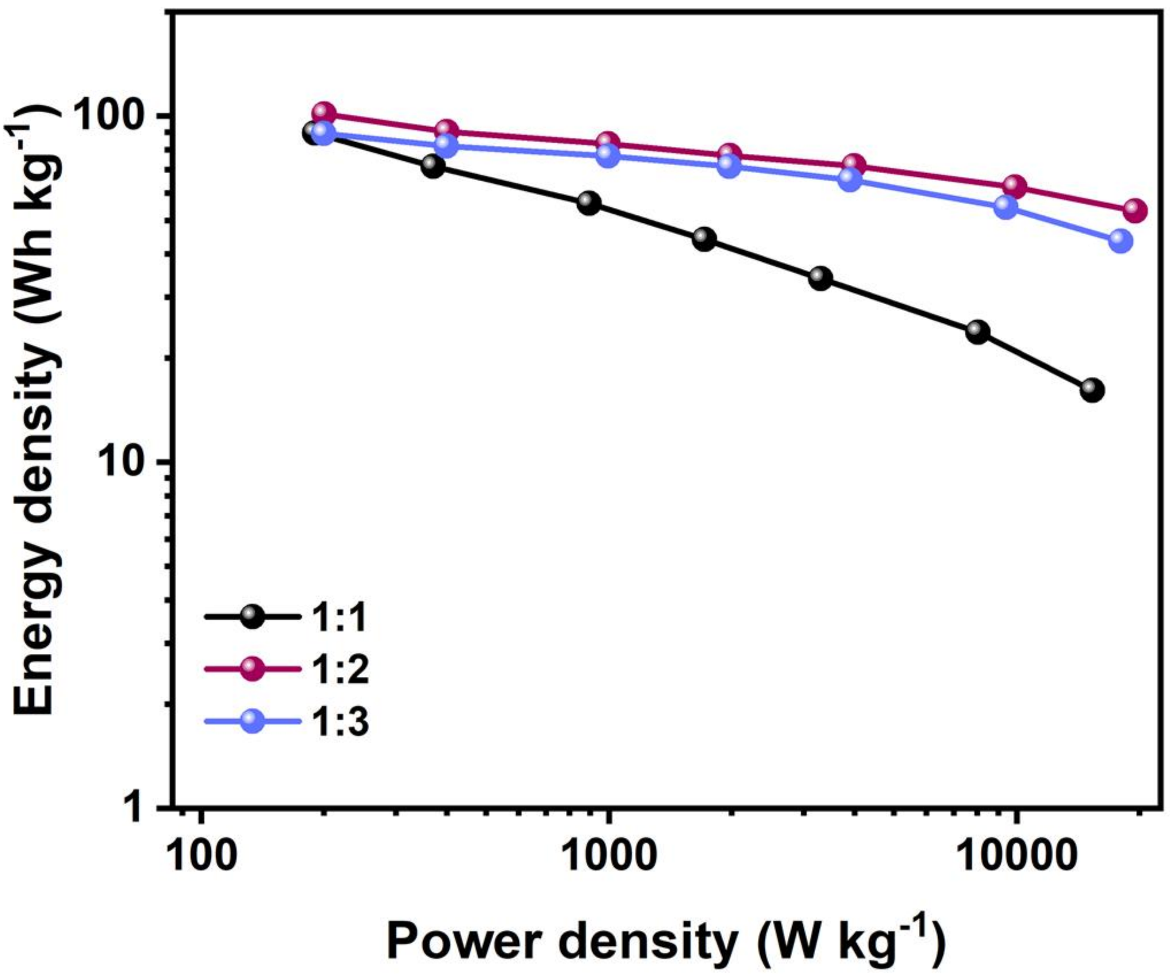

Figure S18. Ragone plot of MDC//K-MDC full cells with the different anode:cathode mass ratios.

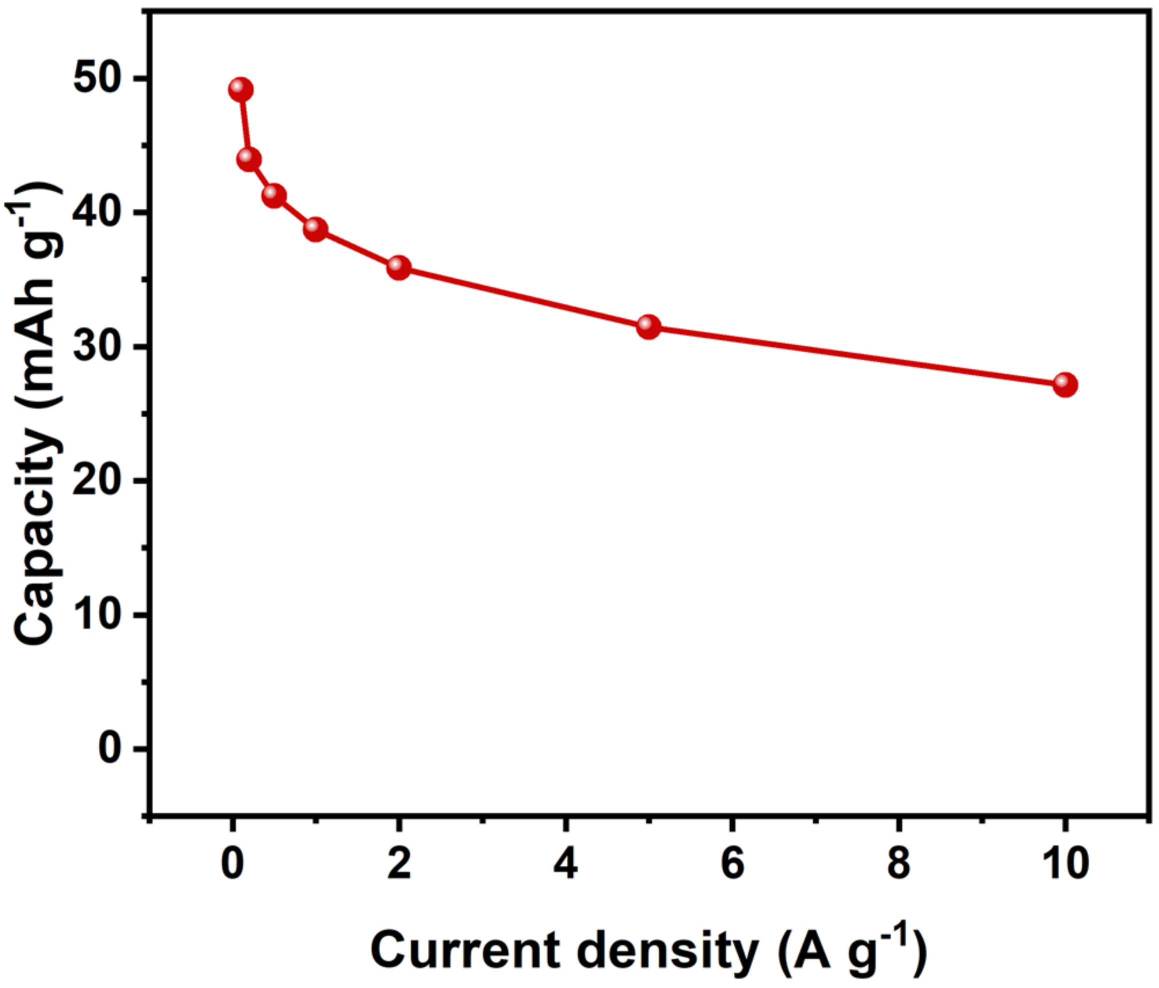

Figure S19. Capacities of MDC//K-MDC full cells at various current densities.

Table S1. Deconvolution of N 1s spectra of MDCs.

Sample	Zn-N (%)	Pyridinic N (%)	Pyrrolic N (%)	Graphitic N (%)	Oxidized N (%)
MDC(0)	1.03	44.63	38.96	6.81	8.57
MDC(20)	14.46	40.29	29.40	8.69	7.16
MDC(50)	24.53	35.95	21.94	7.52	10.06

Table S2. The textural properties of MDCs and K-MDCs.

Sample	S_{BET} (m^2 g^{-1})	V_{total} (cm^3 g^{-1})	V_{micro} (cm^3 g^{-1})	V_{meso} (cm^3 g^{-1})
MDC(0)	1003	0.42	0.38	0.04
MDC(20)	48	0.04	0.00	0.04
MDC(50)	31	0.03	0.00	0.03
K-MDC(0)	2917	1.63	1.07	0.56
K-MDC(20)	5214	2.81	1.70	1.11
K-MDC(50)	3657	2.39	1.10	1.29

Table S3. Surface compositions of K-MDCs.

Sample	Atomic %		
	C	O	N
K-MDC(0)	86.24	11.80	1.96
K-MDC(20)	86.53	11.76	1.71
K-MDC(50)	87.13	11.33	1.54

Table S4. Textural properties of MDC anode.

Sample	S_{BET} (m^2 g^{-1})	V_{total} (cm^3 g^{-1})	V_{micro} (cm^3 g^{-1})	V_{meso} (cm^3 g^{-1})
MDC(anode)	1631	0.70	0.63	0.07

Table S5. Surface compositions of MDC anode.

Sample	Atomic %				
	C	N	O	Zn	Cl
MDC(anode)	76.89	17.78	3.32	1.03	0.99

Table S6. Surface area, reversible capacities, and cycle stability of K-MDC(20) and previous other cathode structures.

Cathode Structures	Surface Area	Reversible Capacity	Cycle Stability (Capacity Retention)	Reference
Chemically activated carbon cathodes				
BAC	1438 m^2 g^{-1}	50 mAh g^{-1} @ 0.5 A g^{-1}	2000 cycles @ 0.5 A g^{-1}	S1
AC	1644 m^2 g^{-1}	79 mAh g^{-1} @ 1 A g^{-1}	500 cycles @ 1 A g^{-1}	S2
Heteroatom-doped carbon cathodes				
NPC-60	1022 m^2 g^{-1}	79 mAh g^{-1} @ 0.1 A g^{-1}	1000 cycles @ 2 A g^{-1}	S3
MnSAs/ NF-CNs	531 m^2 g^{-1}	87 mAh g^{-1} @ 0.1 A g^{-1}	2000 cycles @ 2 A g^{-1} (86%)	S4
K-MDC	5214 m^2 g^{-1}	93 mAh g^{-1} @ 0.1 A g^{-1}	5000 cycles @ 2 A g^{-1} (82%)	This work

Table S7. Energy densities and power densities of MDC//K-MDC full cells at various current densities.

Current density (A g^{-1})	0.1	0.2	0.5	1	2	5	10
Energy density (Wh kg^{-1})	101.3	90.3	83.0	77.0	71.8	62.3	53.2
Power density (kW kg^{-1})	0.2	0.4	1	2	4	10	20

Table S8. Energy densities and power densities of MDC//K-MDC and previously reported dual-carbon SIHCs.

Dual-carbon SIHCs		Maximum Energy density @ Power density	Energy density @ Maximum Power density	Reference
Anode	Cathode			
Graphite	AC	60.5 Wh kg^{-1} @ 1422 W kg^{-1}	21.8 Wh kg^{-1} @ 17127 W kg^{-1}	S5
HAT-CNF-850	STC-16	95 Wh kg^{-1} @ 190 W kg^{-1}	18 Wh kg^{-1} @ 13000 W kg^{-1}	S6
PI/rGO-rGO	rGO	55.5 Wh kg^{-1} @ 395 W kg^{-1}	21.5 Wh kg^{-1} @ 3400 W kg^{-1}	S7
HAT550@ZTC	STC-16	61 Wh kg^{-1} @ 100 W kg^{-1}	12 Wh kg^{-1} @ 24000 W kg^{-1}	S8
GCNF	AC	55.58 Wh kg^{-1} @ 133 W kg^{-1}	18.19 Wh kg^{-1} @ 4520 W kg^{-1}	S9
MDC	K-MDC	101.3 Wh kg^{-1} @ 200 W kg^{-1}	53.2 Wh kg^{-1} @ 20000 W kg^{-1}	This work

Section S3. References

[S1] Y. E. Zhu, L. Yang, J. Sheng, Y. Chen, H. Gu, J. Wei, Z. Zhou, *Adv. Energy Mater.* **2017**, *7*, 1701222.

[S2] S. Liang, S. Zhang, Z. Liu, J. Feng, Z. Jiang, M. Shi, L. Chen, T. Wei, Z. Fan, *Adv. Energy Mater.* **2021**, *11*, 2002600.

[S3] P. Cai, R. Momen, M. Li, Y. Tian, L. Yang, K. Zou, X. Deng, B. Wang, H. Hou, G. Zou, X. Ji, *Chem. Eng. J.* **2021**, *420*, 129647.

[S4] X. Hu, G. Wang, J. Li, J. Huang, Y. Liu, G. Zhong, J. Yuan, H. Zhan, Z. Wen, *Energy Environ. Sci.* **2021**, *14*, 4564.

[S5] X. Liu, G. A. Elia, B. Qin, H. Zhang, P. Ruschhaupt, S. Fang, A. Varzi, S. Passerini, *ACS Energy Lett.* **2019**, *4*, 2675.

[S6] R. Yan, E. Josef, H. Huang, K. Leus, M. Niederberger, J. P. Hofmann, R. Walczak, M. Antonietti, M. Oschatz, *Adv. Funct. Mater.* **2019**, *29*, 1902858.

[S7] Q. Zhao, D. Yang, C. Zhang, X. H. Liu, X. Fan, A. K. Whittaker, X. S. Zhao, *ACS Appl. Mater. Interfaces* **2018**, *10*, 43730.

[S8] R. Yan, K. Leus, J. P. Hofmann, M. Antonietti, M. Oschatz, *Nano Energy* **2020**, *67*, 104240.

[S9] M. L. Divya, S. Jayaraman, Y.-S. Lee, V. Aravindan, *Chem. Eng. J.* **2021**, *426*, 130892.
